# Integrated genetic analyses of immunodeficiency-associated Epstein-Barr virus- (EBV) positive primary CNS lymphomas

**DOI:** 10.1007/s00401-023-02613-w

**Published:** 2023-07-26

**Authors:** Leon D. Kaulen, Evgeniya Denisova, Felix Hinz, Ling Hai, Dennis Friedel, Octavian Henegariu, Dirk C. Hoffmann, Jakob Ito, Alexandros Kourtesakis, Pascal Lehnert, Sofia Doubrovinskaia, Philipp Karschnia, Louisa von Baumgarten, Tobias Kessler, Joachim M. Baehring, Benedikt Brors, Felix Sahm, Wolfgang Wick

**Affiliations:** 1 Department of Neurology, University Hospital Heidelberg, Heidelberg University, Heidelberg, Germany; 2 Clinical Cooperation Unit (CCU) Neuro-Oncology, German Cancer Research Center (DKFZ) and German Consortium for Translational Cancer Research (DKTK), Im Neuenheimer Feld 400, 69120 Heidelberg, Germany; 3 Division of Applied Bioinformatics, German Consortium for Translational Cancer Research (DKTK), German Cancer Research Center (DKFZ), and National Center for Tumor Diseases (NCT), Heidelberg, Germany; 4 Department of Neuropathology, Heidelberg University Hospital, Heidelberg, Germany; 5 Clinical Cooperation Unit (CCU) Neuropathology, German Cancer Research Center (DKFZ) and German Consortium for Translational Cancer Research (DKTK), Heidelberg, Germany; 6 Department of Neurosurgery, Yale School of Medicine, New Haven, USA; 7 Department of Genetics, Yale School of Medicine, New Haven, USA; 8 Faculty of Biosciences, Heidelberg University, Heidelberg, Germany; 9 Department of Neurosurgery, Munich University Hospital, Ludwig Maximilians University (LMU) Munich, and German Cancer Consortium (DKTK) Partner Site, Munich, Germany; 10 Department of Neurology, Yale School of Medicine, New Haven, USA

**Keywords:** Primary CNS lymphoma, Non-Hodgkin lymphoma, Epstein-Barr virus, Genetics, Exome sequencing, RNA sequencing, Immunodeficiency

## Abstract

Immunodeficiency-associated primary CNS lymphoma (PCNSL) represents a distinct clinicopathological entity, which is typically Epstein-Barr virus-positive (EBV^+^) and carries an inferior prognosis. Genetic alterations that characterize EBV-related CNS lymphomagenesis remain unclear precluding molecular classification and targeted therapies. In this study, a comprehensive genetic analysis of 22 EBV^+^ PCNSL, therefore, integrated clinical and pathological information with exome and RNA sequencing (RNASeq) data. EBV^+^ PCNSL with germline controls carried a median of 55 protein-coding single nucleotide variants (SNVs; range 24–217) and 2 insertions/deletions (range 0–22). Genetic landscape was largely shaped by aberrant somatic hypermutation with a median of 41.01% (range 31.79–53.49%) of SNVs mapping to its target motifs. Tumors lacked established SNVs (MYD88, CD79B, PIM1) and copy number variants (CDKN2A, HLA loss) driving EBV^−^ PCNSL. Instead, EBV^+^ PCNSL were characterized by SOCS1 mutations (26%), predicted to disinhibit JAK/STAT signaling, and mutually exclusive gain-of-function NOTCH pathway SNVs (26%). Copy number gains were enriched on 11q23.3, a locus directly targeted for chromosomal aberrations by EBV, that includes SIK3 known to protect from cytotoxic T-cell responses. Losses covered 5q31.2 (STING), critical for sensing viral DNA, and 17q11 (NF1). Unsupervised clustering of RNASeq data revealed two distinct transcriptional groups, that shared strong expression of CD70 and IL1R2, previously linked to tolerogenic tumor microenvironments. Correspondingly, deconvolution of bulk RNASeq data revealed elevated M2-macrophage, T-regulatory cell, mast cell and monocyte fractions in EBV^+^ PCNSL. In addition to novel insights into the pathobiology of EBV^+^ PCNSL, the data provide the rationale for the exploration of targeted therapies including JAK-, NOTCH- and CD70-directed approaches.

**Supplementary Information:**

The online version contains supplementary material available at 10.1007/s00401-023-02613-w.

## Introduction

Primary central nervous system lymphoma (PCNSL) represents a rare extranodal variant of non-Hodgkin lymphoma (NHL) with an incidence of 0.45/100,000 [49]. Per definition, the disease is restricted to the central nervous system (CNS) at diagnosis including the brain parenchyma, spinal cord, meninges and eyes [31, 57].

Primary (e.g., genetic syndromes) or secondary (e.g., administration of immunosuppressants, human immunodeficiency virus (HIV) infection) immunodeficiency increases the risk of CNS lymphomagenesis and accounts for 5–10% of cases. These immunodeficiency-associated PCNSL constitute a distinct clinicopathological entity, which was recognized in the 2021 World Health Organization classification of CNS tumors [39]. Unlike disease in immunocompetent hosts, cases are typically (85–95%) Epstein-Barr virus-positive (EBV^+^), suggesting an EBV-related pathogenesis, and remain associated with an inferior outcome [9, 15, 26, 33, 47]. This may reflect either the inability to tolerate high-dose methotrexate-based polychemotherapy and/or EBV-driven oncogenesis with peculiar molecular properties. A better pathogenetic understanding of EBV^+^ PCNSL is hence necessary to pave the way for novel targeted therapies.

In recent years sequencing studies have helped to uncover the genetic landscape of immunocompetent, EBV^−^ PCNSL [7, 11, 20, 30, 32, 50, 67]. Tumors predominantly harbor alterations in the B-cell receptor (BCR), toll-like receptor (TLR), and nuclear factor kappa B (NF-kB) signaling pathways. This includes PIM1, MYD88 L265P and CD79B Y196 hotspot mutations detected in 50–100%, 50–85% and 36–63% of cases, respectively. Genetics and tumor microenvironment of 44 EBV^+^ PCNSL were recently assessed using targeted sequencing and digital multiplex gene expression [21]. Tumors lacked hotspot mutations typically found in EBV^−^ disease and were characterized by elevated macrophage and immune-checkpoint gene expression in HIV-negative and low CD4 gene counts in HIV-infected individuals. While this suggested a distinct molecular landscape, alterations that characterize EBV-related CNS lymphomagenesis remained unclear precluding further molecular classification and development of targeted therapies.

In this study, a comprehensive genetic analysis of 22 EBV^+^ PCNSL was therefore performed, which integrated clinical and pathological information with whole exome (WES) and RNA sequencing (RNASeq) data. This allowed us to uncover the protein-coding genetic landscape of EBV^+^ PCNSL and led to the identification of alterations, that may guide targeted treatment approaches of this rare entity.

## Methods

### Cohort and sample collection

Retrospective case review identified 19 treatment-naïve immunocompromised adult EBV^+^ PCNSL, collected at the Department of Neuropathology of University Hospital Heidelberg, with sufficient tissue for molecular characterization. Steroids were avoided in the two weeks before surgical diagnosis. Diagnosis was re-confirmed by a board-certified neuropathologist in all cases prior to study entry. Per histomorphological assessment, the tumor cell content exceeded 60% in all samples. Slit lamp examination and lumbar punctures were negative for ocular and cerebrospinal fluid involvement at diagnosis. Systemic disease was ruled out using fluorodeoxyglucose positron emission tomography or whole-body computed tomography as well as bone marrow biopsy. Retrospective chart review provided additional clinical and radiological data.

The discovery WES cohort included five fresh-frozen (FF) EBV^+^ samples with matching blood (germline) controls. Given the rarity of the disease, this cohort was supplemented with analyzed genetic data from three previously reported EBV^+^ cases that had matching blood controls (YNHH-6, LS-GD-0111, LS-GD-0106) [21, 32]. The in-house WES extension cohort consisted of FF or formalin-fixed paraffin-embedded (FFPE) tissue from 11/14 EBV^+^ samples, where matching blood controls were unavailable. Additionally, WES was carried out on two random EBV^−^ PCNSL to confirm our pipeline detected previously established alterations. RNA sequencing (RNASeq) was performed on 19/19 EBV^+^ specimen from our institution, including all 16 in-house cases that also underwent WES.

### Immunohistochemistry

Immunohistochemical stainings were performed on a Ventana BenchMark ULTRA Immunostainer (Ventana Medical Systems, Tucson, USA) using FFPE sections. The panel included CD20, CD10, BCL6, CD3, MUM1/IRF4, LMP1, PD-L1 and Ki67. Details on antibodies are provided in the supplementary. Samples were classified into germinal center B-cell-like (GCB) and non-GCB subtypes according to the Hans algorithm (BCL6, CD10, MUM1) [29].

### EBV status assessment

EBV status was evaluated with immunohistochemistry (EBV-LMP1) and Epstein-Barr encoding region (EBER) in-situ hybridization following previously described protocols [16, 21]. Following previous studies, only cases where EBV was detected in more than half of the tumor cells were included in this study.

### DNA and RNA extraction

Genomic DNA and RNA were extracted using the Promega Maxwell RSC device (Promega, Madison, USA). DNA was isolated with the Maxwell 16 Tissue DNA purification kit, the Maxwell 16 blood purification kit, or the Maxwell 16 FFPE Plus LEV DNA purification kit (Promega, Madison, USA) according to the manufacturer’s instructions. RNA was extracted with the Maxwell 16 LEV simplyRNA or the Maxwell 16 LEV RNA FFPE purification kit (Promega, Madison, USA) following the manufacturer’s protocols. Agilent 4200 TapeStation (Agilent Technologies, Santa Clara, USA) was used to determine RNA integrity numbers.

### Whole exome sequencing and processing

Exome capture was performed with the Agilent SureSelect All Exon V7 kit (Agilent Technologies, Santa Clara, USA). The libraries were paired-end sequenced on an Illumina NovaSeq 6000 or NextSeq 500 (Illumina, San Diego, USA).

#### Alignment of reads

Mapping and preprocessing were performed in the DKFZ OTP (One Touch Pipeline) system using DKFZ AlignmentAndQCWorkflow version 1.2.73-202 (https://github.com/DKFZ-ODCF/AlignmentAndQCWorkflows). Briefly, reads were mapped to the human reference genome build hs37d5 (phase II reference of the 1000 Genomes Project including decoy sequences) using the Burrows-Wheeler Aligner (BWA) version 0.7.15 mem function with all default parameters, except minimum base quality threshold which was set to zero (−T 0) [5, 35]. BAM files were sorted using SAMtools version 0.1.19, and PCR duplicates were marked using Sambamba version 0.6.5 [36, 63].

#### SNVs

Single nucleotide variants (SNVs) were detected using an in-house workflow (https://github.com/DKFZ-ODCF/SNVCallingWorkflow) based on SAMtools/BCFtools with parameter adjustments to allow for somatic variant calling and heuristic filtering as previously described in the ICGC Pan-Cancer Analysis of Whole Genome project [65]. For paired samples, SNV calling was performed in OTP using SNVCallingWorkflow version 1.2.166-3 based on SAMtools/BCFtools version 0.1.19. For tumor samples without matched controls, SNVCallingWorkflow version 2.1.1-0 based on SAMtools/BCFtools version 1.9 was run in a no-control mode. No-control mode strategy included additional filters which removed common SNPs and recurrent artifacts using variant frequency information from public and local control sample pools. The local control pool contained variant frequency from 4879 whole genome (WGS) and 1198 whole exome (WES) samples analyzed with the same workflows. Variants with minor allele frequency (MAF) above 0.01 in 1000 Genomes or 0.001 in gnomAD (WGS or WES) or with a frequency above 0.01 in the local control pool (WGS or WES) were annotated as common SNPs or artifacts and removed from the downstream analysis [5]. To remove artifacts a 'confidence score' was calculated for each mutation. This score was initially set to 10 and subsequently reduced if the mutation overlapped with genomic regions that are known to be prone to artifacts. Those regions have been identified using the following UCSC genome browser tracks (http://genome.ucsc.edu/cgi-bin/hgTables [52]): GENCODE Mappability track, UCSC27 High Seq Depth track, UCSC Simple-Tandemrepeats, UCSC Repeat-Masker, DUKE-Excluded, DACBlacklist, UCSC Selfchain. High-confidence SNVs were defined as having a score of at least 8. Variants were annotated with GENCODE v19 using ANNOVAR (version 2016Feb01) [18, 70]. Only somatic non-silent coding variants (i.e., nonsynonymous, stopgain, stoploss, or splicing in a vicinity of 2 bp of exon boundaries) of high confidence were selected (except for the analysis of mutational signatures, where all high confidence, including non-coding and silent, somatic variants from the capture regions were used).

#### INDELs

Short insertions/deletions (indels) were identified using in-house workflow (https://github.com/DKFZ-ODCF/IndelCallingWorkflow) based on Platypus (version 0.8.1.1) as previously described [65]. For paired samples, indel calling was performed in OTP using IndelCallingWorkflow version 2.4.1-1. For tumor samples without matched controls, IndelCallingWorkflow version 3.1.0-0 was run in a no-control mode. As in the SNV workflow, the no-control mode strategy included additional filters based on variant frequency information from public and local control sample pools. A scoring scheme like that applied in the SNV workflow was used. Indels were annotated with GENCODE v19 using ANNOVAR and somatic high-confidence indels (minimum confidence of 8) falling into a coding sequence or splice site were selected for the analysis.

#### Copy number alterations (CNAs)

Copy number alterations (CNAs) and loss of heterozygosity regions of in-house samples were identified using a workflow based on the software package CNVkit version 2.1.0 with default parameter setting [61]. For tumor samples without matched controls, same-gender control samples from other patients were used as “artificial controls”. Tumor cell content and ploidy were estimated using a method adapted from ACEseq (https://aceseq.readthedocs.io/en/latest/. Segments with a total copy number (TCN) at least 0.7 above the tumor ploidy were defined as gains and segments with a TCN at least 0.7 below it were defined as losses. High-level CNAs were defined as homozygous deletions and amplifications (> 2.5 × the average ploidy). CNA analysis in FFPE samples is challenging and may result in artifacts, particularly with respect to copy number losses. To address this, GISTIC2.0 workflow with default parameters (except for *q* = 0.1 and log2 ratio amplification/deletion threshold = 0.3/− 0.3) was used to identify regions of significantly recurring gains or losses [44]. Segmented log2 ratios (.cns) were generated by CNVkit and converted to a segmentation.seg file. This file was then used as input for the GISTIC2.0 module.

#### Mutational signature

Mutational signature contributions were calculated using the R/Bioconductor package YAPSA (Yet Another Package for Signature Analysis, https://bioconductor.org/packages/release/bioc/html/YAPSA.html); the Alexandrov-COSMIC (AC) signatures (https://cancer.sanger.ac.uk/cosmic/signatures_v2) [2, 3].

#### Viral integration detection

The presence of viral DNA and genomic integration in tumor samples was detected using an in-house pipeline based on VIRUSBreakend [8]. Starting with an aligned BAM file, it identifies viral reads of interest through Kraken2 taxonomic classification of all unaligned or partially aligned sequences using a custom Kraken2 database [73].

### RNA sequencing (RNASeq)

#### Sequencing, processing, and alignment

Illumina TruSeq stranded mRNA kit was used to prepare RNASeq libraries. Sequencing was performed on the Illumina HiSeq X Ten V2.5 platform (Illumina, San Diego, USA). Paired-end reads were aligned to the human genome (hg19) using STAR (v2.5.2b) with default settings. Sambamba (v0.4.6) marked duplicate reads. SAMtools (v1.19) allowed coordinate sorting of BAM files. Gene-specific reads were quantified with the human gene model (GENCODE v19) using featureCounts in Subread (v1.5.1) with default settings. Gene read count matrix was obtained for further analyses.

#### Unsupervised clustering analysis

The top 500 highly variable genes were selected for hierarchical clustering analysis. The optimal number of clusters in hierarchical clustering was selected based on the Silhouette method using R package factoextra (v1.0.7). Three clusters were obtained (i.e., EBV_1, EBV_2 and EBV_3).

#### Differential expression (DE) analysis

Pairwise DE analyses between three clusters (i.e., EBV_1 vs. EBV_2, EBV_2 vs. EBV_3 and EBV_1 vs. EBV_3) were performed in R package DESeq2 (v1.32.0) [40]. Genes with total counts < 10 in all samples were excluded. Genes were normalized with variance stabilizing transformation (vst) method. DE results were shrunk using apeglm. The top 50 DE genes (DEGs) in comparisons with adjusted *p* value < 0.05 and log2 fold change > 2 (upregulated DEGs) or log2 fold change < 2 (downregulated DEGs) were selected as features for clusters. For example, EBV_1 included the top 50 upregulated DEGs and the top 50 downregulated DEGs in EBV_1 vs. EBV_2 and EBV_1 vs. EBV_3. Expression clusters were compared with the gene read count matrix of healthy brain tissue RNA-Seq data downloaded from the Genotype-Tissue Expression (GTEx) Portal (https://www.gtexportal.org/home/). The seven samples (Supplementary Table 5) were randomly selected and included various brain areas from both genders.

#### Gene ontology (GO) enrichment analysis

The enriched GOs for each cluster were obtained using R package clusterProfiler (v4.0.5) with biological process Gos from the org.Hs.eg.db database. The top 10 Gos with *q* value < 0.05 were visualized in bar plots using ggplot2 (v3.3.5).

#### Deconvolution of bulk RNA Sequencing

Deconvolution of bulk RNA Sequencing (counts-per-million [CPM] values) from our EBV^+^ and a previously published EBV^−^ cohort from the ICGC-MML-Seq consortium [50] were performed using CIBERSORTx (https://cibersortx.stanford.edu) [48]. The signature matrix of human hematopoietic cell phenotypes (LM22) defined immune cell subsets [12]. The imputed cell fractions were visualized in box plots using ggpubr (v0.4.0).

### Validation of sequencing

Selected variants (SOCS1, NOTCH1, KMT2D, IGH-V169) were validated using Sanger Sequencing of tumor and blood control samples. For validation of copy number variants (CNVs) and RNASeq data we subjected genomic and synthesized complementary DNA to quantitative PCR, respectively. Details are provided in the supplementary.

### Ethics

This study was approved by the Heidelberg University Medical Faculty institutional review board (S-128/2022). Written informed consent was obtained from all study participants or their legal guardians. The study was conducted in accordance with the Declaration of Helsinki.

## Results

### Cohort characteristics

The study cohort included 8 paired and 14 unpaired treatment-naïve immunodeficiency-related EBV^+^ PCNSL. All cases were classified as diffuse large B-cell lymphoma (DLBCL). The cell-of-origin was determined according to the Hans algorithm with most lymphomas attributed to the ABC subtype (*n* = 20). Clinical/pathological cohort characteristics are summarized in Supplementary Table 1.

### Exome sequencing

Exome sequencing was performed with a median coverage of 226X (range 41–526) and 294X (range 248–321) on in-house tumor and blood specimen, respectively. Sequencing data was assessed for the presence of viral genetic material. EBV sequences (NC_007605.1) were detected in all included EBV^+^ cases, matching pathological evaluations. No overlap with viral sequences was found in both EBV^−^ tumors (Supplementary Fig. 1), that carried established SNVs (e.g., CD79B p.Y196H, PIM1, TBL1XR, HLA-B, HLA-C) and CNVs (e.g., CDKN2A and HLA loss).

#### Exonic variant counts

**Fig. 1:**
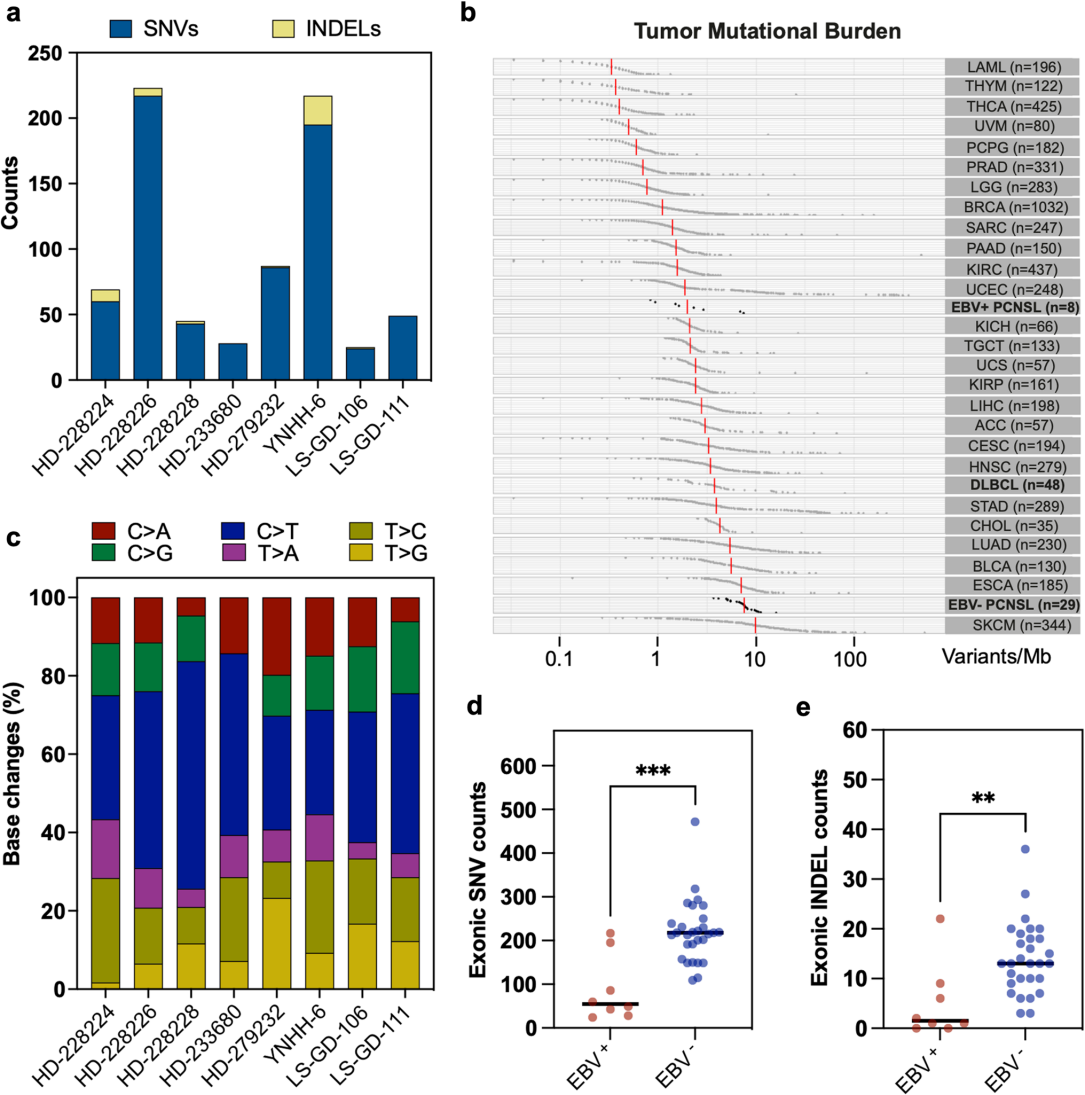
Single nucleotide variant and insertion/deletion counts. **a** Functional exonic single nucleotide variants (SNVs, blue) and insertions/deletions (INDELs, yellow) counts are displayed. Paired EBV^+^ PCNSL carried a median of 55 protein-coding SNVs (range 24–217) and 2 INDELs (range 0–22). **b** Tumor mutational burden (TMB: variants/megabase (Mb)) for various malignancies deposited in the cancer genome atlas (TCGA) are shown and contrasted to EBV^+^ PCNSL from this study and EBV^−^ counterparts from the ICGC MMML-Seq consortium. TMB in EBV^+^ PCNSL was lower than in systemic lymphomas (DLBCL) and EBV^−^ PCNSL. **c** Base substitutions were conserved across cases and indicate a C > T predominance in agreement with the deamination of cytosines. **d** EBV^+^ PCNSL carried significantly (Mann–Whitney test; ***p < 0.001; **p < 0.01) fewer non-synonymous exonic SNV and INDELs (**e)** than EBV^−^ PCNSL from the ICGC MMML-Seq consortium

In the paired EBV^+^ PCNSL samples (*n* = 8, Fig. 1a), a median of 55 functional, protein-coding SNVs (range 24–217) and a median of 2 INDELs (range 0–22) were found. Base transitions revealed a C > T predominance across samples in agreement with deaminated unmethylated cytosines (Fig. 1c). Median tumor mutational burden (TMB) was 1.9/megabase (Mb) (Fig. 1b). Comparison to other tumor entities deposited in the cancer genome atlas (TCGA) revealed median TMB in immunocompetent systemic DLBCL patients was higher (3.8/Mb). Similarly, according to a recently published dataset [50], TMB in EBV^−^ PCNSL (7.5/Mb) exceeded EBV^+^ counterparts included in this study. This was reflected by a significantly higher number of functional exonic SNVs (*p* < 0.001, Fig. 1d) and INDELs (*p* < 0.01, Fig. 1e) in EBV^−^ PCNSL.

#### Aberrant somatic hypermutation

**Fig. 2:**
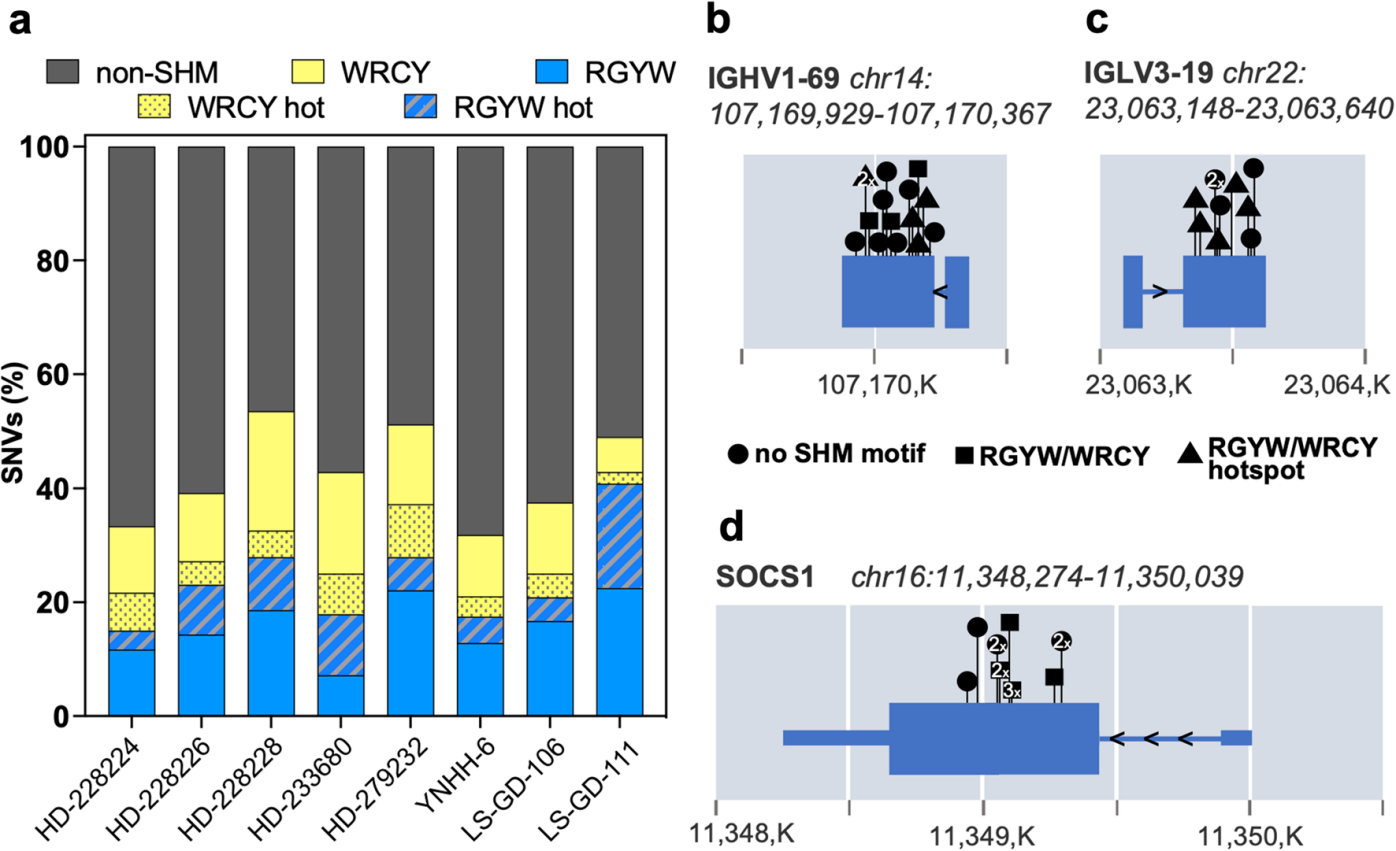
Aberrant somatic hypermutation EBV^+^ PCNSL. **a** SNV fractions that overlapped with aberrant somatic hypermutation target motifs WRCY (yellow) and RGYW (blue) were determined (W = A/T; R = A/G; C or G = hotspot; Y = C/T). A median 41.01% of SNVs localized to either motif, suggesting a critical role for aSHM in the pathobiology of EBV^+^ PCNSL. **b**, **c**, **d** Genes with alterations within the first 2000 (*K* = 1000) bases from the transcriptional start site, that frequently mapped to aSHM motifs, are shown. Positions of detected variants are indicated with symbol shapes denoting relation to target motifs. Targets in EBV^+^ PCNSL included immunoglobulin genes (**b**, **c**), but also SOCS1 (**d**)

Genetic landscape of EBV^−^ PCNSL is largely shaped by aberrant somatic hypermutation (aSHM), which is driven by activation-induced cytidine deaminase (AID) enzyme-mediated cytosine deamination [32, 50, 67]. It targets the WRCY sequence motif (W = A/T; R = A/G; C = hotspot; Y = C/T) and its reverse complement, RGYW. To assess the relevance of aSHM in EBV^+^ disease, the fraction of SNVs that overlapped with its target motifs was determined in our paired cohort (Fig. 2a). A median of 41.01% (range 31.79–53.49%) of SNVs localized to the WRCY (median: 17.5%, range 8.16–25.58%) or RGYW (median 21.94%, range 15–40.82) motifs. The WRCY and RGYW motif hotspots were targeted in 4.41% (range 2.04–9.3%) and 7.28% (range 3.33–18.37%), respectively. Despite the high overlap of SNVs with aSHM motifs, paired EBV^+^ tumors lacked PIM1 variants, the most prominent target of aSHM in EBV^−^ PCNSL [50, 67]. In addition to somatic hypermutation of immunoglobulin genes (Fig. 2b, c), SOCS1 SNVs frequently mapped to aSHM motifs (Fig. 2d) and were found within the first 2000 bases from the transcriptional start site.

#### Altered genes in EBV^+^ PCNSL

**Fig. 3:**
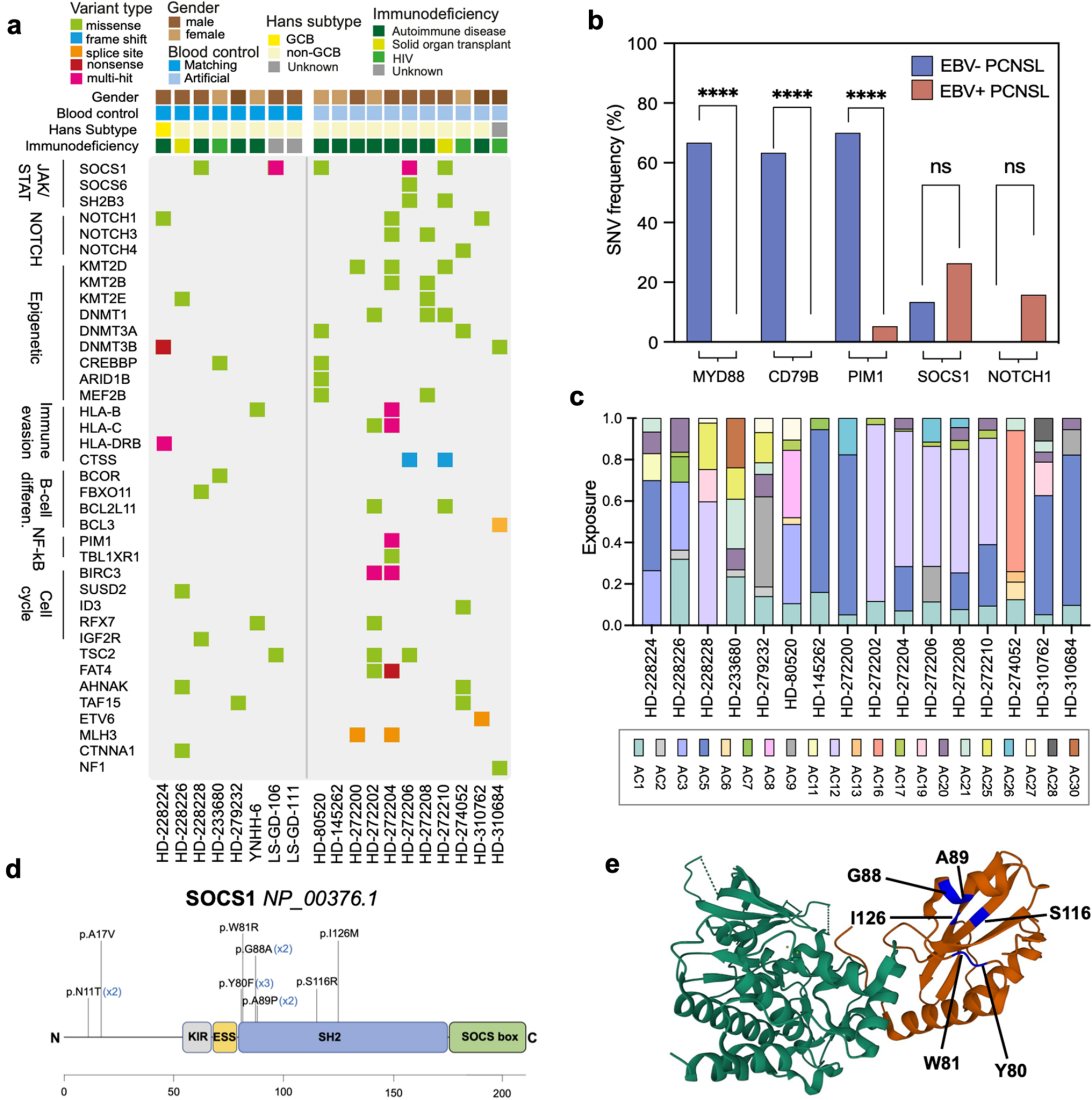
Single nucleotide variants in EBV^+^ PCNSL. **a** Filtered genes with functional exonic SNVs and INDELs are shown. Colors indicate the type of alteration. SOCS1 was the most frequently (26%, *n* = 5) altered gene in EBV^+^ PCNSL. Mutually exclusive NOTCH variants were detected in 26% (*n* = 5) of tumors. **b** Frequencies of selected alterations are contrasted to EBV^−^ PCNSL (blue) from the ICGC-MMML-Seq dataset. EBV^+^ cases (red) mostly lacked MYD88, CD79B, and PIM1 variants characteristic of virus-negative disease (blue). Frequencies were compared with Fisher Exact tests and significance levels are displayed (****p < 0.0001; ns = not significant). **c** Normalized single base substitution Alexandrov-COSMIC (AC) mutational signatures in EBV^+^ PCNSL are shown. Colors indicate the signature type and proportions reflect related SNVs counts/sample. Signatures associated with spontaneous deamination of cytosines (AC1), and defective DNA mismatch repair (AC20, AC21) were frequently found. **d** The SOCS1 gene and its domains are displayed (KIR, kinase inhibitory region; ESS, extended SH2 subdomain; SH2, Src2 homology domain; SOCS box) and detected alterations are indicated. Most (*n* = 10/13) variants clustered to the SH2 domain, which mediates binding and subsequent inhibition of JAK1. **e** Crystal structure of the SOCS1 SH2 domain (red) in complex with the JAK1 kinase domain (green) obtained from the RCSB protein data bank (accession number 6C7Y). Positions of altered amino acids (blue) in EBV^+^ PCNSL are indicated within the complex

Detected SNVs were filtered for relevance in tumorigenesis (Fig. 3a) using the COSMIC Gene Census database and the network of cancer genes catalogue of known and candidate cancer genes (NCG.v7). Immunoglobulin gene SNVs, which constitute a feature of healthy and neoplastic B-cells resulting from somatic hypermutation, were found in all cases. EBV^+^ PCNSL lacked known MYD88 p.L265P and CD79B p.Y196 hotspot mutations, which were previously described to drive CNS lymphomagenesis in EBV^−^ cases (Fig. 3b) [7, 11, 20, 30, 32, 50, 67]. PIM1 and TBLXR1, two frequently altered genes in EBV^−^ PCNSL such as in our control samples, were altered in a single unpaired case only (HD-272204). Overall, except for the above-mentioned case, EBV^+^ PCNSL harbored few alterations in the BCR and NF-kB signaling pathways.

#### JAK/STAT pathway

As a target of aSHM, the most frequently altered gene in EBV^+^ PCNSL was SOCS1 with heterozygous variants detected in five cases (26%) including two with germline controls (Fig. 3a). Most hits (*n* = 10/13) clustered within the SH2 domain (Fig. 3d, e), which mediates binding to JAK1, critical for inhibition of the JAK/STAT pathway [38]. This included recurrent variants p.Y80F (*n* = 3), p.G88A (*n* = 2), and p.A89P (*n* = 2). Several alterations (p.N11T, p.A17V, p.W81R, p.G88A, p.A89P, p.S116R) were annotated in the COSMIC database and previously reported in systemic DLBCL. MutationTaster predicted p.N11T, p.Y80F, p.W81R, p.A89P, p.S116R, and p.I126M were deleterious. Bioinformatic loss-of-function predictions were additionally corroborated by two samples carrying multiple non-synonymous SNVs and matched previously suggested tumor suppressive properties of SOCS1 in lymphoma.

Heterozygous germline SOCS1 variants were previously linked to severe autoimmunity with aberrant lymphoproliferation and transition to lymphomas in some cases [27]. Blood controls from this study were therefore assessed for additional SOCS1 alterations. Second hits in cases with somatic SNVs were absent. However, a germline SOCS1 variant of unknown significance (p.Q210H, rs11549428) was found in one individual with autoimmune disease-related PCNSL. This variant is found at a population frequency of 0.18% according to the 1000 Genomes Project, lies outside the SH2 domain and could also represent a benign polymorphism.

Somatic variants were detected in additional inhibitory JAK/STAT pathway genes, namely SOCS gene family member SOCS6 (p.K65I) as well as adaptor protein SH2B3 (p.D69N). MutationTaster predicted deleterious effects for variants further suggesting disinhibition of the JAK/STAT pathway.

#### NOTCH pathway

NOTCH SNVs occurred in five EBV^+^ PCNSL (26%). NOTCH1 variants were uncovered in three specimens (16%) including one paired case (Fig. 3a). Two of three variants localized to heterodimerization (p.R1598H) and PEST (p.H2500L) domains, respectively, that carry known hotspots for gain-of-function mutations [71]. SNVs in the heterodimerization domain were previously shown to expose the metalloprotease S2 cleavage site resulting in ligand-independent cleavage, and release of the intracellular receptor domain with subsequent pathway activation. Alterations in the PEST domain prevent ubiquitin-mediated proteasomal degradation of the activated receptor. MutationTaster prognosticated pathogenic consequences for detected NOTCH1 SNVs. Additional hits in the NOTCH pathway included NOTCH3 (*n* = 2, 11%) and NOTCH4 variants (*n* = 1, 5%). NOTCH3 p.G1422A was found in the proximity of the heterodimerization domain whereas NOTCH4 p.R1924W localized to the PEST domain suggesting gain-of-function. Of note, NOTCH and SOCS1 SNVs were mutually exclusive in EBV^+^ PCNSL.

*Epigenetic regulators*, which are commonly mutated in EBV^−^ disease, also carried several SNVs in EBV^+^ PCNSL (Fig. 3a). Overall, variants in respective genes were detected in eleven cases (58%). They co-occurred with JAK/STAT or NOTCH pathway alterations. Affected genes included lysine methyltransferases KMT2 genes (KMT2D *n* = 3; KMT2B *n* = 2; KMT2E: *n* = 2), DNA-methyl-transferase (DNMT) genes (DNMT1 *n* = 3; DNMT3A *n* = 2; DNMT3B *n* = 2), and lysine acetyltransferase CREBBP (*n* = 2) among others.

#### Mutational signatures

Detected variants were compared with previously described Alexandrov-COSMIC (AC) mutational signatures (Fig. 3c) [2, 3]. Patterns associated with spontaneous deamination of 5-methylcytosine (AC1), which is enriched in EBV^−^ PCNSL [50], were frequently detected in EBV^+^ PCNSL. Variants were also linked to AID/APOBEC family of cytidine deaminases activity (AC2). Overlap with signatures related to defective DNA mismatch repair (AC20, AC21) and homologous recombination DNA mismatch repair (AC3) were frequently identified. Additionally, mutational patterns of unknown etiology were found (AC12, AC17).

#### CNAs

**Fig. 4:**
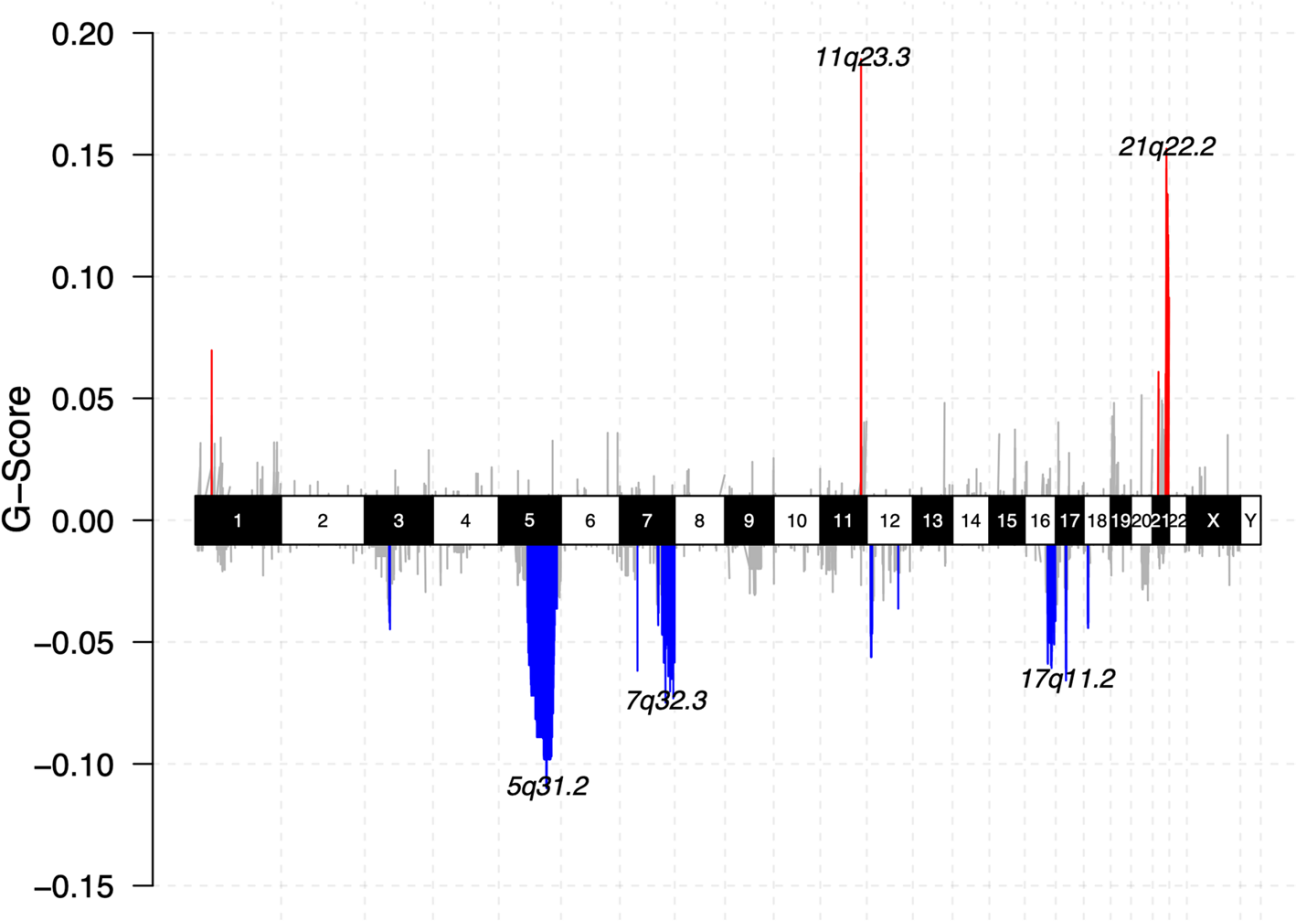
Copy number alterations in EBV^+^ PCNSL. Chromoplot summarizes copy number variants (CNVs) of in-house EBV^+^ PCNSL. Chromosomes are displayed on the *x*-axis. The G-score, which integrates frequency across samples and the magnitude of CNVs, is shown on the *y*-axis. Gains (red) and losses (blue) are indicated. Lymphomas carried 11q23, a site known to be specifically targeted for genomic aberrations by EBV, and 21q22 gains. Losses covered 5q31, the locus of STING1 necessary for sensing of viral cytoplasmic DNA, 7q32 and 17q11

A median of 6.5% (range 2.5–14.7%) of the genome were affected by CNAs (Fig. 4). CDKN2A or HLA loss, characteristic for EBV^−^ PCNSL, were not found EBV^+ ^cases. Frequent gains were found on 11q23.3 (FOXR1, SIK3), a site known to be directly targeted for genomic aberrations by EBV protein, EBNA1 [37]. Additionally, gains included 21q22.2 (ERG) and NOTCH1. The latter were also found in samples, which lacked additional NOTCH pathway SNVs, providing an alternative mechanism of pathway activation. Losses were enriched on 5q31, the locus of STING1 critical for sensing viral DNA with subsequent induction of apoptosis [46]. Further losses were identified on 7q32 (IRF5), and 17q11 (NF1).

### RNA sequencing

#### Expression clusters

**Fig. 5:**
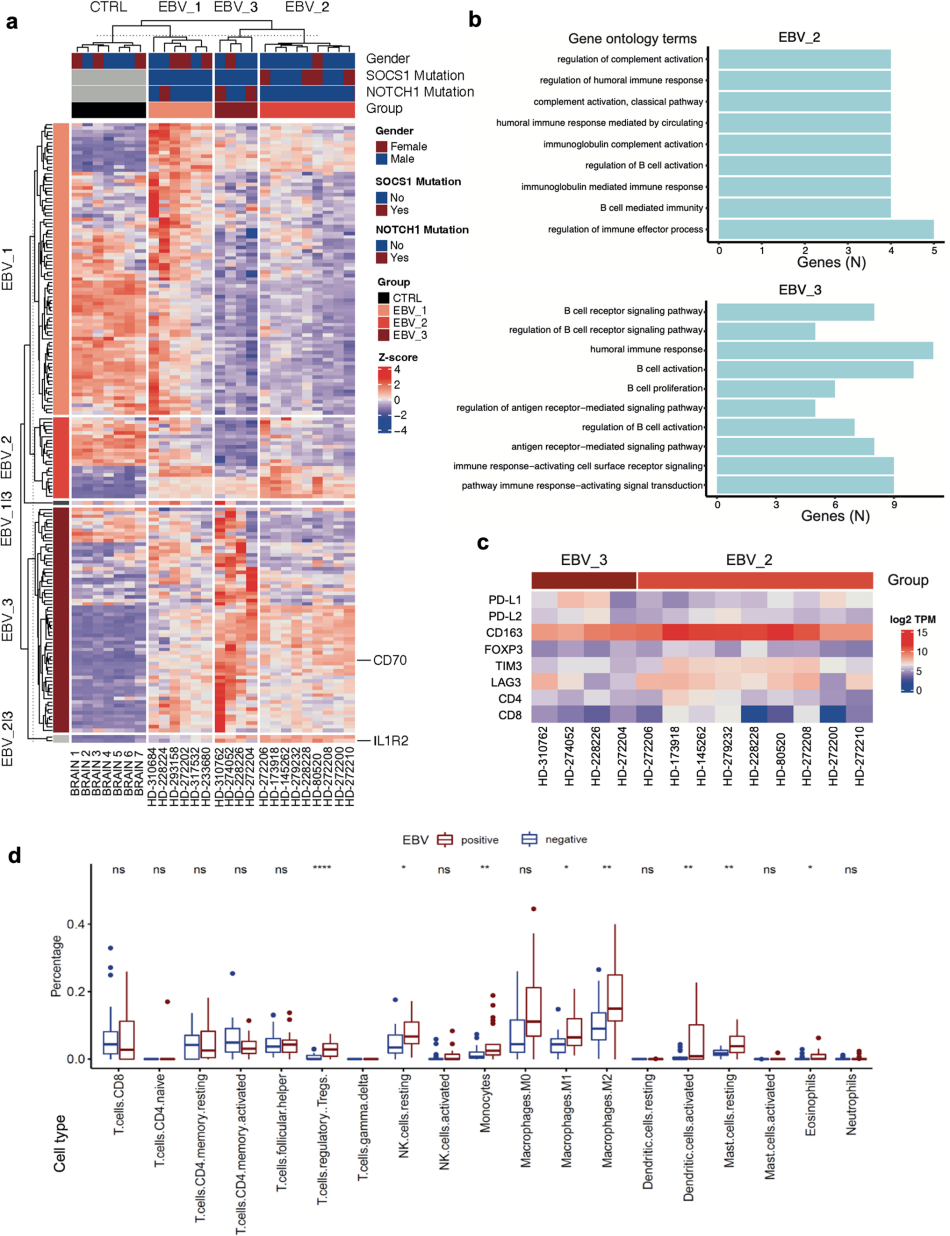
Expression clusters and tumor microenvironment in EBV^+^ PCNSL. **a** Heatmap shows scaled expression levels of feature genes from three EBV^+^ PCNSL expression groups, identified with unsupervised hierarchical clustering analysis, across samples and healthy brain controls from the GTEx database. Cluster 1 (EBV_1) showed high overlap with GTEx controls in line with healthy brain contamination and cases were removed from further analyses. Clusters 2 (EBV_2) and 3 (EBV_3) separated lymphomas with SOCS1 and NOTCH1 SNVs and displayed robust IL1R2 and CD70 expression. **b** Top 10 enriched gene ontology (GO) terms for feature genes from these clusters are shown. GOs were ordered with rising q values from top to bottom and number (N) of cluster genes overlapping with respective GO terms are indicated along the x-axis. GOs largely reflected B-cell-related terms. **c** Heatmap indicates robust expression of immune checkpoint genes and markers of a tolerogenic tumor microenvironment (TME) across EBV^+^ PCNSL. **d** Correspondingly, deconvolution of bulk RNASeq data (CIBERSORTx) from EBV^+^ PCNSL and EBV^−^ cases from the ICGC-MMML-Seq dataset revealed a tolerogenic TME with enrichment of T-regulatory cell, M2-macrophage, monocyte, and mast cell fractions in EBV-related tumors (Mann–Whitney test; ****p < 0.0001; ***p < 0.001, **p < 0.01, *p < 0.05; *ns* not significant)

Unsupervised hierarchical clustering revealed three expression clusters in EBV^+^ PCNSL (Fig. 5a). As normal brain contamination was encountered in previous studies performing RNA-sequencing on PCNSL specimen [50], DEGs were compared with expression patterns from healthy brain controls deposited in GTEx. DEGs found in cluster 1 largely reflected brain tissue expression patterns. This was further corroborated by enriched gene ontology (GO) terms (Supplementary Fig. 3) and cases were excluded from further analyses. In contrast, clusters two and three shared B-cell related terms including ‘regulation of B-cell activation’, or ‘regulation of immune effector processes’ (Fig. 5b). Of note, cases with SOCS1 alterations were found within expression cluster 2, whereas tumors with NOTCH1 SNVs mapped to cluster 3.

Expression groups 2 and 3 shared robust IL1R2 and CD70 expression. Corresponding to strong expression in EBV^+^ PCNSL, analysis of the GTEx portal database revealed CD70 overexpression in EBV-transformed B-lymphocytes (median 340.6 transcripts per million, Supplementary Fig. 4). Significantly DEGs between both clusters included stronger immunoglobulin constant gene and MAPK4 expression in cluster 2 whereas TRAF4 and SMO were among upregulated genes in group 3 (Supplementary Fig. 5).

#### CIBERSORTx analysis suggests a tolerogenic tumor microenvironment

In addition to immune checkpoints (PD-L1/L2, LAG3, TIM-3) strongly expressed IL1R2 and CD70 can inhibit immune responses and create a tolerogenic tumor microenvironment (TME) [41, 64]. We found strong expression of CD163 (M2-macrophages) and FOXP3 (T-regulatory cells, Treg; Fig. 5c) across EBV^+^ PCNSL. To further characterize the TME, deconvolution of bulk RNA sequencing data was performed using CIBERSORTx (Fig. 5d) comparing our EBV^+^ dataset with previously published EBV^−^ PCNSL [50]. In agreement with a tolerogenic TME significant enrichment of Tregs, M2-macrophages, monocytes and mast cells were found in EBV^+^ PCNSL.

## Discussion

In this study, comprehensive genetic analyses of 22 EBV^+^ PCNSL integrated clinical and pathological data with WES and RNASeq. This provided novel mechanistic insights into EBV-driven CNS lymphomagenesis and revealed alterations, that may serve as diagnostic markers and/or may guide targeted therapy.

Previous work suggested EBV^+^ PCNSL lack hotspot mutations detected in EBV^−^ PCNSL [11, 32]. This was further supported by a recent study [21], that characterized 44 EBV^+^ PCNSL FFPE samples using targeted sequencing (54 genes) and a gene expression panel (14 genes). In agreement with our WES findings, SNVs (PIM1, MYD88, CD79B) and CNVs (CDKN2A, HLA loss) characteristic of EBV^−^ disease were absent. Corresponding to fewer alterations within their sequencing panel, WES now revealed a lower TMB in EBV^+^ PCNSL, which parallels other EBV^+^ lymphomas outside the nervous system [22]. However, alterations that drive EBV^+^ PCNSL remained unclear given their study design, and top hits from this WES/RNASeq study (Fig. 3 SNVs: SOCS1, NOTCH; Fig. 4 CNVs: 11q23.3 gain, 5q31.2 loss; Fig. 5 RNA: CD70, IL1R2) were not part of their panel.

The genetic landscape of NHL is largely shaped by aSHM driven by AID activity [32, 50, 67]. As EBV^+^ PCNSL lacked variants in most prominent targets including PIM1, its influence on the genetic landscape was unclear. Although target genes differed, the high overlap of SNVs with aSHM motifs, however, suggested a critical role during EBV-driven CNS lymphomagenesis. This mirrors other EBV-related tumors such as Burkitt lymphoma, where the genetic landscape is largely shaped by AID activity and subsequent aSHM [24]. Underlining its pathogenetic relevance in EBV^+^ PCNSL, like PIM1 in EBV^−^ tumors the most frequently mutated gene in EBV^+^ disease, SOCS1, represents a known target of aSHM and several detected SNVs clustered within its target motifs [45].

The suppressor of the cytokine signaling (SOCS) gene family including its most potent member, SOCS1, provides an inducible negative feedback loop for cytokine-triggered JAK/STAT pathway activation [69]. SOCS1 directly interacts with JAK1 via its SH2 domain, which results in ubiquitination and subsequent proteasomal degradation of JAK1 as well as direct inhibition of Janus kinase activity [38]. Most detected variants in EBV^+^ PCNSL localized to the SH2 domain and bioinformatic scores predicted loss-of-function effects pointing to JAK/STAT pathway disinhibition. SOCS1 mutations were also documented in EBV^−^ PCNSL albeit reported at lower frequencies [30, 50]. Whether SOCS alterations are indeed enriched in EBV^+^ disease warrants further investigation given the available case numbers. However, detection in other EBV-related lymphomas (systemic DLBCL including HIV-related disease; Hodgkin lymphoma) supports their relevance during EBV-driven lymphomagenesis [10, 22, 72]. Of note, JAK/STAT activation is known to drive the expression of EBV oncogenes including LMP1 via direct interaction of STAT molecules with promoter regions in EBV-related tumors [13, 14]. Additionally, the pathway up-regulates immune checkpoint PD-L1, strongly expressed in EBV^+^ PCNSL, and may hence contribute to a tolerogenic microenvironment [25]. SOCS1 variant-related disinhibition of the pathway may allow to unleash the viral oncogenic potential. Modeling of similar SOCS1 variants—including SH2 domain alterations—in EBV-transformed B-lymphocytes and HEK293 cells previously revealed excessive JAK/STAT pathway activation upon cytokine stimulation [27]. This sensitized for treatment with the JAK1/JAK2 inhibitor ruxolitinib, which is approved for the treatment of myeloproliferative neoplasms and was shown to penetrate the blood–brain barrier in mice [28, 68]. These findings provide a rationale for preclinical and early clinical assessment of JAK inhibition in EBV^+^ PCNSL.

In addition to JAK/STAT pathway variants, mutually exclusive NOTCH SNVs were detected in EBV^+^ PCNSL. Most alterations localized to mutational hotspots in the heterodimerization and PEST domains, which result in gain-of-function in T-cell and chronic lymphocytic leukemia [53, 71]. Additional NOTCH1 amplifications provide an alternative mechanism and further support genetic pathway activation in EBV^+^ PCNSL. This matches EBV^+^ systemic lymphomas, where activating NOTCH2 SNVs were detected [22, 58], which are rare in virus-negative tumors. NOTCH activation was previously shown to maintain oncogenic viral latency transcriptional programs and prevent lytic cycles and subsequent cell death in EBV^+^ tumors, which was reverted with NOTCH inhibitor treatment [23]. Additionally, NOTCH drives immune evasion via induction of PD-L1 and could contribute to a tolerogenic microenvironment in EBV^+^ PCNSL [42]. Various NOTCH inhibitors are under ongoing preclinical/clinical development and evaluation in EBV^+^ PCNSL may be warranted based on alterations identified in this study [4].

Frequent detection of variants in epigenetic regulators was in line with the previous targeted sequencing approach, which identified KMT2D/KMT2C as top hits in EBV^+^ PCNSL although mutational frequencies were lower than in EBV^−^ tumors [21]. However, they were among the few typical variants shared between virus-positive and -negative lymphomas. KMT2D, the most frequently altered epigenetic modifier in EBV^+^ PCNSL, also represented the top hit in a CRISPR-Cas9 screen investigating DLBCL drivers [51]. This may suggest that epigenetic regulators are implicated in CNS lymphomagenesis irrespective of EBV status.

Distinct CNV profiles were identified in EBV^+^ PCNSL, which overall may impair antiviral defense mechanisms and promote B-cell activation. This included 11q23 gains, the locus of SIK3, which protects cells from cytotoxic T-cell responses [59]. Of note, this locus is specifically and directly targeted for genomic aberrations by the EBV protein, EBNA1 [37]. An 11q23 gain/loss pattern was previously found in posttransplant Burkitt-lymphoma-like disease [54], whereas tumors in immunodeficient hosts from this cohort lacked respective deletions. Additionally, loss of STING1 (5q31.2) may hamper cytoplasmic viral DNA sensing and disinhibit BCR signaling [46, 62, 74]. 7q32 deletions cover IRF5, a known tumor suppressor in B-cell lymphoma, while individuals with NF1 loss are at increased risk for NHL [6, 60].

Transcriptional profiling revealed two distinct clusters in EBV^+^ PCNSL, that separated cases with SOCS1 and NOTCH1 SNVs. In addition to previously reported robust PD-L1 expression [11, 21], both expression groups shared strong IL1R2 and CD70 expression, which may shape a tolerogenic TME. IL1R2 constitutes a decoy interleukin receptor, that abrogates interleukin-1 signaling and is known for strong expression in serum and tumor tissue in EBV-related Hodgkin lymphoma [41]. CD70 belongs to the TNF receptor superfamily and is predominantly expressed on activated B- and T-cells. Via interaction with its receptor, CD27, it acts as an immune checkpoint that can co-stimulate B-cell activation, but also inhibit immune responses via T-cell exhaustion/apoptosis [17, 64]. Overexpression in tumors was linked to more aggressive phenotypes and allowed immune evasion via a tolerogenic TME. Given the absence of expression in most healthy tissue, CD70 is evaluated as a novel immunotherapeutic target and CD70-directed antibodies as well as CAR T-cells showed promising efficacy in preclinical but also early clinical studies [17, 56]. This study revealed CD70 as an interesting candidate for targeted therapy in EBV^+^ PCNSL. Further membrane proteins that shared robust expression in EBV^+^ tumor samples and low/absent levels in most healthy tissues included PD-L1, PD-L2, LAG3, TIM3 as well as B-cell marker CD19. As a result, these genes carry the potential for immunotherapeutic approaches, some of which have already been explored in EBV^−^ PCNSL as well as in other malignancies [19, 34]. However, immunocompromised patients were excluded from most clinical trials due to concerns for immune-related adverse events (irAE). While a growing body of evidence shows that immunotherapy can be safely administered in many immunodeficient patients and most irAE are manageable, an individual risk–benefit analysis as well as vigilant monitoring in a referral center setting are warranted [1, 43, 66].

In agreement with the overexpression of immune checkpoint genes, RNA-sequencing suggested a tolerogenic TME including the presence of Tregs (FOXP3) and particularly M2-macrophages (CD163). Similarly, CD163 was previously reported significantly up-regulated in EBV^+^ PCNSL compared to EBV^−^ counterparts [21]. Deconvolution of bulk RNASeq datasets in this study further revealed higher M2-macrophages, Treg, monocyte and mast cell fractions in EBV^+^ PCNSL. This is in agreement with other EBV-related lymphomas (e.g. Hodgkin), where immune evasion via a tolerogenic TME contributes to virus-driven lymphomagenesis [55].

Limitations of this study include the cohort size, which reflects the rarity of EBV^+^ PCNSL and/or the lack of tissue availability. However, sequencing studies in more prevalent EBV^−^ disease enrolled similar or even lower case numbers [7, 11, 20, 32, 67]. Germline controls were also not available for the entire cohort, which was addressed with a rigorous filtering approach based on recurrent and previously reported alterations. Supplemented, previously described paired cases were processed using different workflows, which will have introduced batch effects. Yet high similarity to in-house paired samples including detection of recurrent SOCS1 and NOTCH alterations additionally supported our findings. Strengths of this study include the integrated WES/RNASeq approach, which led to the identification of novel variants and markers in EBV^+^ PCNSL (e.g., SOCS1, NOTCH1, CD70) and suggested a tolerogenic TME. In addition to insights into the pathomechanism of this rare entity this provides the rational for the exploration and development of new targeted therapies. This is particularly important as many EBV^+^ patients are unable to tolerate aggressive methotrexate-based polychemotherapy resulting in an inferior outcome. Previous functional studies together with bioinformatic scores provide a good body of evidence for the likely functional relevance and/or targetability of identified alterations. Yet further in vitro and in vivo characterization should be considered in future studies to allow rapid translation of our findings from *bench to bedside*. Additionally, a future immunogenomics approach (e.g. HLA typing, neoepitope prediction with subsequent experimental validation) could allow us to identify further targets for immunotherapy.

## Conclusions

The genetic landscape of EBV^+^ PCNSL is largely shaped by aSHM although tumors lacked variants characteristic of EBV^−^ disease. Lymphomas frequently harbored SNVs in the JAK/STAT and NOTCH signaling pathways while CNVs point to impaired antiviral defense mechanisms. Transcriptional profiling revealed two distinct expression clusters, which shared robust expression of immune checkpoint genes including CD70 and IL1R2. Correspondingly deconvolution of bulk RNASeq data suggested a tolerogenic tumor microenvironment. Findings pave the avenue for the (pre-)clinical evaluation of several targeted therapies including JAK/STAT and NOTCH inhibition as well as CD70-directed treatments.

## Supplementary Information

Below is the link to the electronic supplementary material.

Supplementary file1 (DOCX 5928 KB)

## Data Availability

WES and bulk RNASeq were deposited in European Genome-phenome Archive (accession: *EGAS00001007222*). Data access can be obtained from the listed Data Access Committee upon reasonable request. Remaining data are provided within this article and its supplementary. All codes are publicly available.
