# Supplementary material for: Integrated genetic analyses of immunodeficiency-associated Epstein-Barr virus- (EBV) positive primary CNS lymphomas
Source: Acta Neuropathol. 2023 Jul 26;146(3):499–514. doi: 10.1007/s00401-023-02613-w (PMC10412493; doi:10.1007/s00401-023-02613-w)
Supplement: Supplementary file 1 — Supplementary file1 (DOCX 5928 KB) [file 401_2023_2613_MOESM1_ESM.docx]

**Supplementary Data**

**Supplementary Table 1:** Clinical and pathological cohort characteristics

| Clinical  characteristics | Paired cohort (n=8) | (%) | Extension cohort  (n=14) | (%) | Entire cohort  (n=22) | (%) |
| --- | --- | --- | --- | --- | --- | --- |
| Median age, y  [range] | 56  [34-81] |  | 62  [43-81] |  | 62  [34-81] |  |
| Female | 2 | (25) | 5 | (36) | 7 | (32) |
| Male | 6 | (75) | 9 | (64) | 15 | (68) |
| Immunodeficiency | | | | | | |
| Autoimmune disease | 4 | (50) | 10 | (71) | 14 | (64) |
| Solid organ transplantation | 1 | (13) | 2 | (14) | 3 | (14) |
| HIV | 1 | (13) | 2 | (14) | 3 | (14) |
| Unknown | 2 | (25) | 2 | (14) | 4 | (18) |
| Immunosuppressants | | | | | | |
| Azathioprine | 3 | (60) | 6 | (55) | 9 | (56) |
| MMF | 3 | (60) | 6 | (55) | 9 | (56) |
| Cyclosporine | 1 | (20) | 0 | (0) | 1 | (6) |
| Median interval to diagnosis, mo [range] | 70  [33-129] |  | 29  [3-143] |  | 34  [3-143] |  |
| Pathological characteristics | | | | | | |
| DLBCL | 8 | (100) | 14 | (100) | 22 | (100) |
| GCB subtype | 1 | (13) | 0 | (0) | 1 | (5) |
| Non-GCB subtype | 7 | (87) | 13 | (93) | 20 | (91) |
| - N/A | 0 | (0) | 1 | (7) | 1 | (5) |
| PD-L1 expression (IHC) | 3 | (75) | 11 | (100) | 14 | (93) |
| *Abbreviations*: *WES, whole exome sequencing; y, years; HIV, human immunodeficiency virus; MMF, mycophenolate mofetil; mo, months; DLBCL, diffuse large B-cell lymphoma; GCB, germinal center B-cell; N/A, not available; PD-L1, programmed death-ligand 1* | | | | | | |

**Supplementary Fig. 1.** SNVs and CNVs of in-house EBV^-^-PCNSL

**
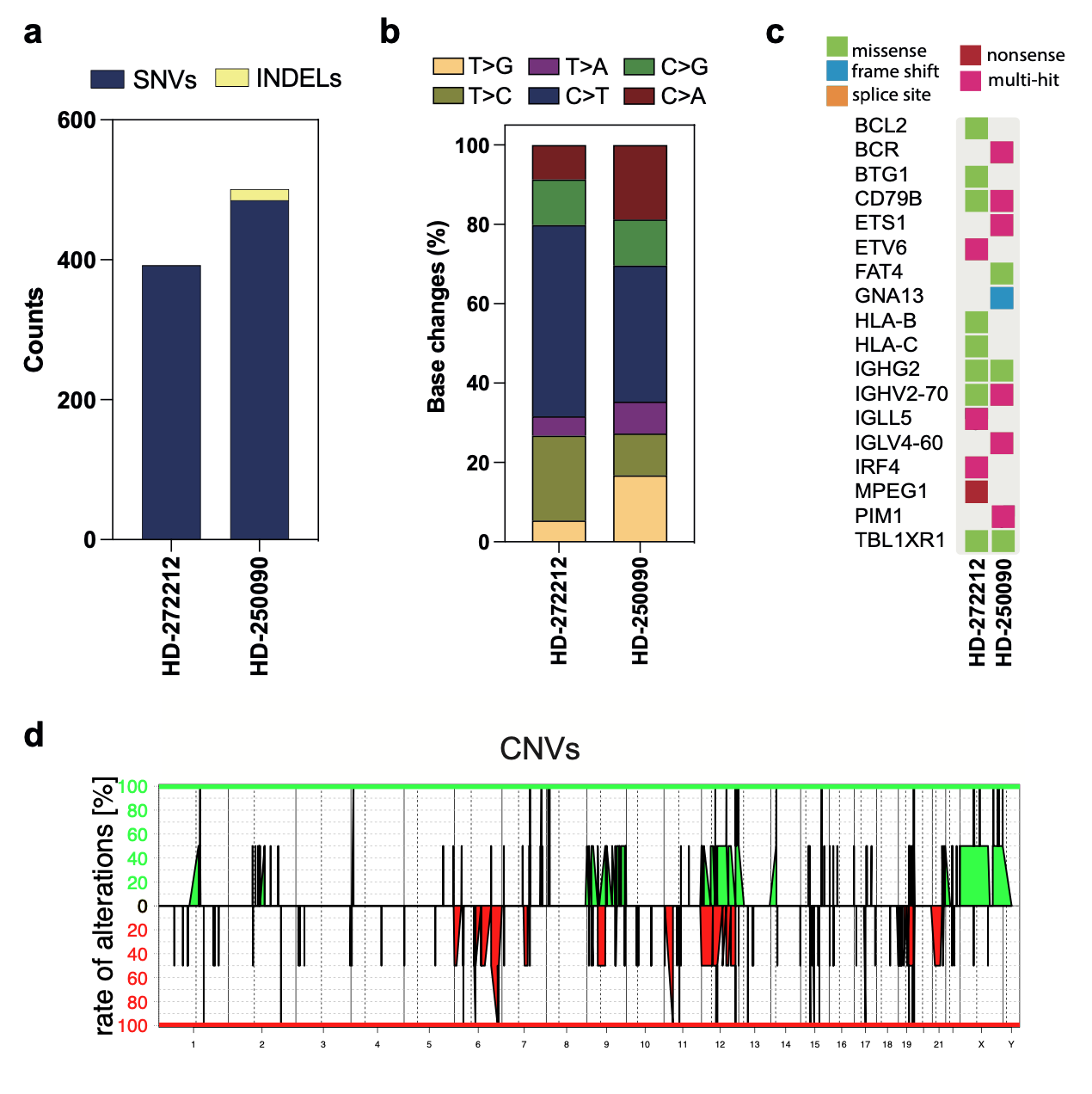
Legend:** WES was performed on two EBV^-^-PCNSL to validate our pipelines. **a** Exonic single nucleotide variant (SNV) and insertion/deletion (INDEL) counts per sample are shown. **b** Base changes revealed a C>T predominance in both cases in agreement with previous studies. **c** SNVs and INDELs in genes previously implicated in CNS lymphomagenesis are shown. Colors encode variant types. Typical PIM1 and CD79B SNVs were detected in both EBV^-^-PCNSL. Additionally somatic hypermutation of immunoglobulin genes was found. **d** Copy number gains (green) and losses (red) are shown. The y-axis indicates frequencies of respective alterations while chromosomes are displayed along the x-axis. Both tumors carried CDKN2A loss (9p21), which is found in most EBV^-^-PCNSL, detected in both in-house cases. Homozygous deletion of the HLA gene locus on chromosome 6 was found in one case.

**Supplementary Fig. 2.** Representative histological findings in EBV+ PCNSL


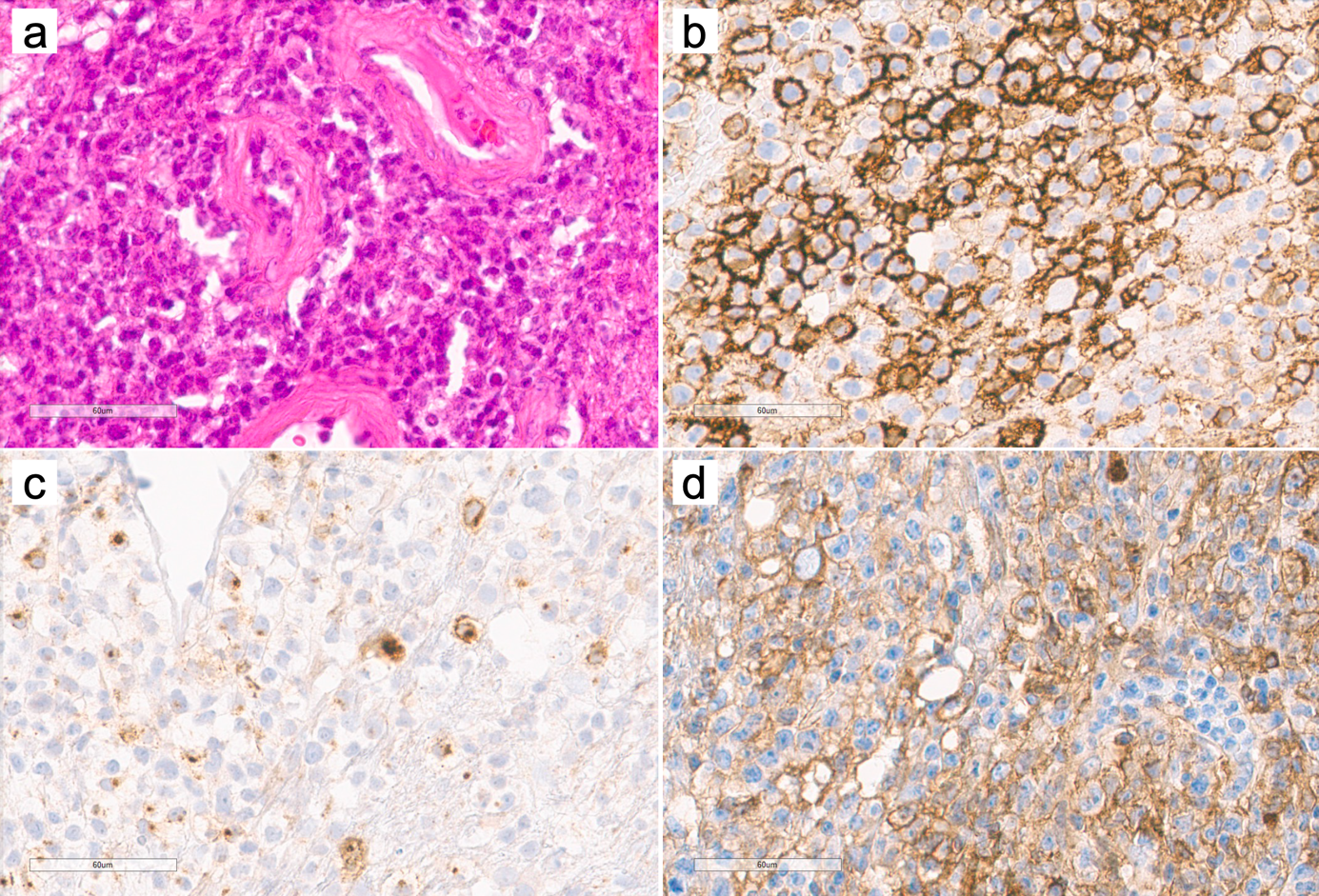


**Legend: A** Hematoxylin and eosin staining at diagnosis of EBV^+^-PCNSL reveals a pleomorphic lymphoid infiltrate. **b** Immunohistochemical staining reveals CD20 positivity, Epstein-Barr virus (EBV-LMP, **c**) is detected within tumor cells and strong PD-L1 expression is found (**d**). Original magnification x400 and scale bar depicts 60μm.

**Supplementary Fig. 3.** GO terms enriched in expression cluster 1.

**Legend:** The top 10 enriched gene ontology (GO) terms for the feature genes of EBV cluster_1 are shown. GOs were ordered with rising q values from top to bottom. Number (N) of cluster genes overlapping with respective GO terms are shown along the x-axis. In agreement with high overlap with healthy brain expression patterns, enriched GO terms reflect normal brain contamination in cluster 1.

**Supplementary Fig. 4.** CD70 tissue expression


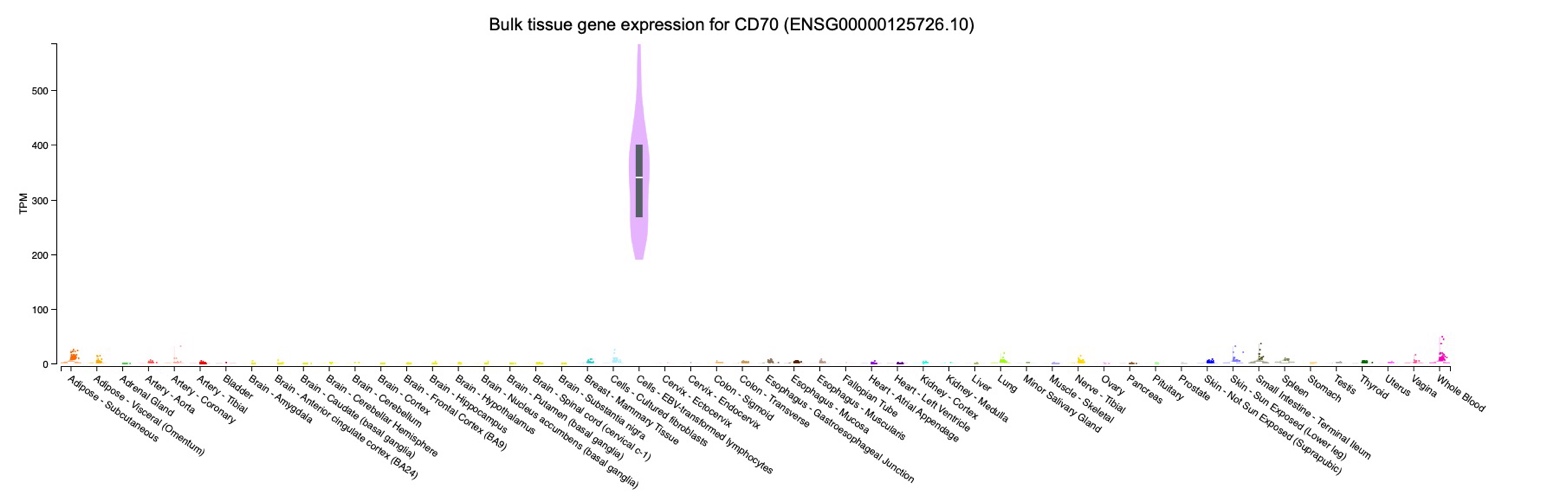


**Legend:** The Genotype-Tissue Expression (GTEx) portal was assessed for CD70 expression across deposited bulk tissue gene expression datasets (GTEx Analysis Release V8 (dbGaP Accession phs000424.v8.p2). With a median of 340.6 transcripts per million (TPM) expression was highest in EBV-transformed lymphocytes. Expression was absent or nearly absent in other tissues. The second highest expression was found in spleen samples with a low median of 2.5 TPM. The figure was created with the GTEx portal website (<https://gtexportal.org/>).

**Supplementary Fig. 5.** Selected DEGs in expression clusters 2 and 3

**
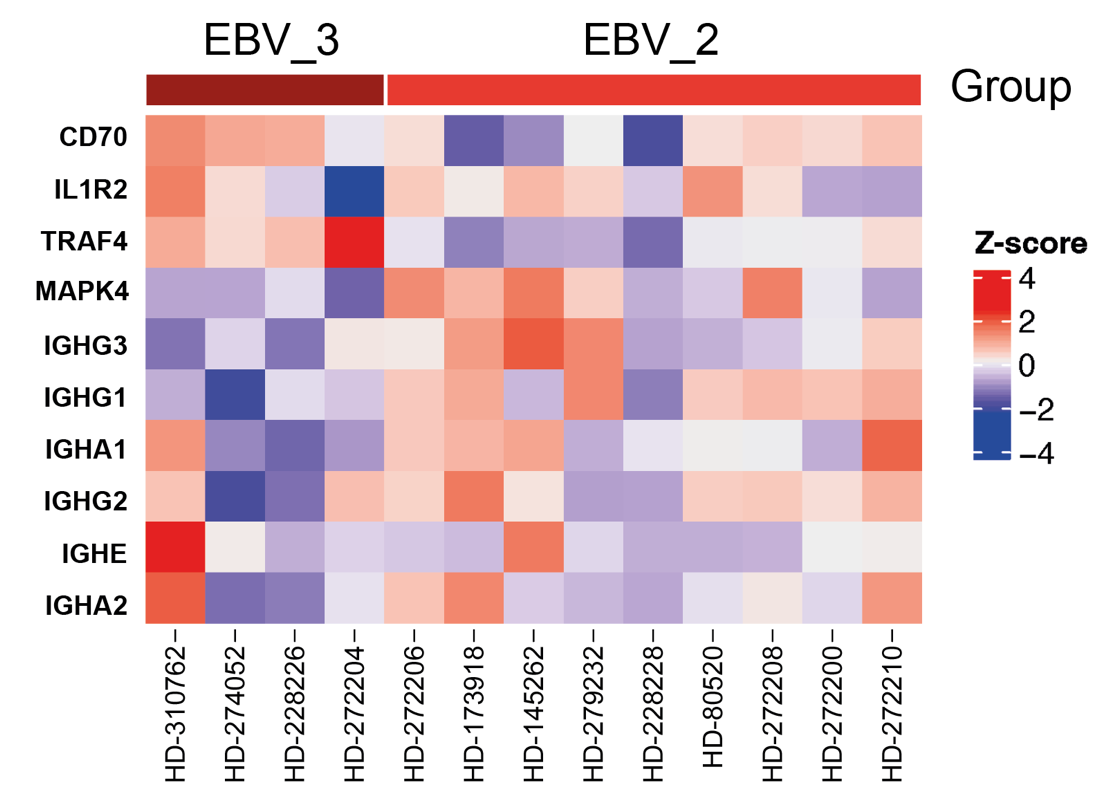
**

**Legend:** Heatmap shows normalized expression of selected marker genes from expression clusters 2 and 3. Expression clusters 2 and 3 share IL1R2 and CD70 expression. Cluster 2 is characterized by significant upregulation of MAPK4 and several immunoglobulin constant genes whereas expression group 3 carries higher TRAF4 levels.

**Supplementary Fig. 6.** SOCS1 variants– Sanger sequencing


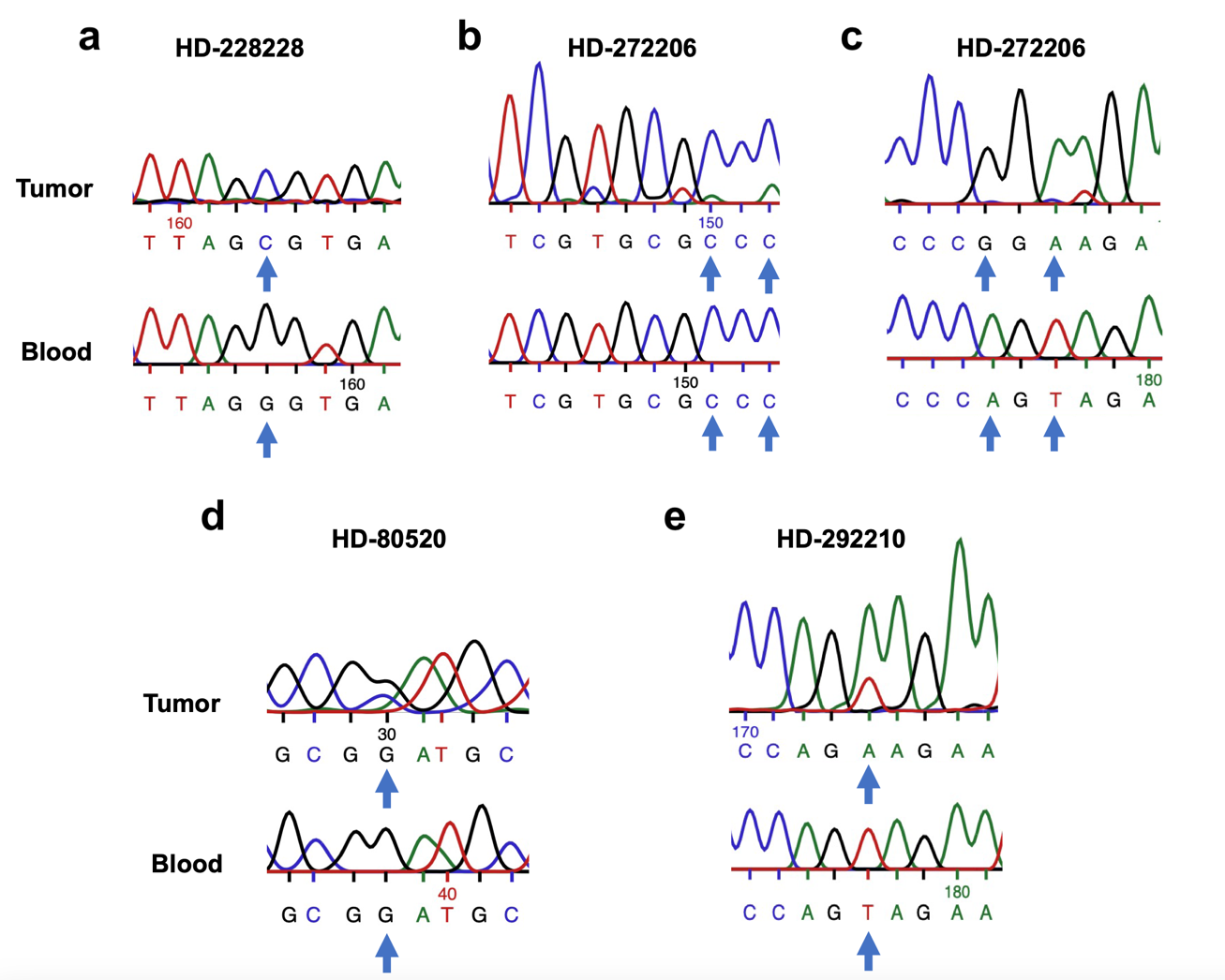


**Legend:** Sanger sequencing results from tumor and blood (germline) specimen are shown. Samples without a corresponding germline control were matched to artificial blood controls from our study that were predicted to be wildtype based on WES. Sample identifiers and variant positions (arrows) are indicated. **a** SOCS1:c.C348G:p.S116R **b** SOCS1:c.G265C:p.A89P and SOCS1:c.G263C:p.G88A **c** SOCS1:c.T241C:p.W81R; and SOCS1: c.A239T:p.Y80F **d** SOCS1:c.378G;p.I126M **e** SOCS1:c.A239T:p.Y80F.

**Supplementary Fig. 7.** IGHV1-69 variants – Sanger sequencing

**
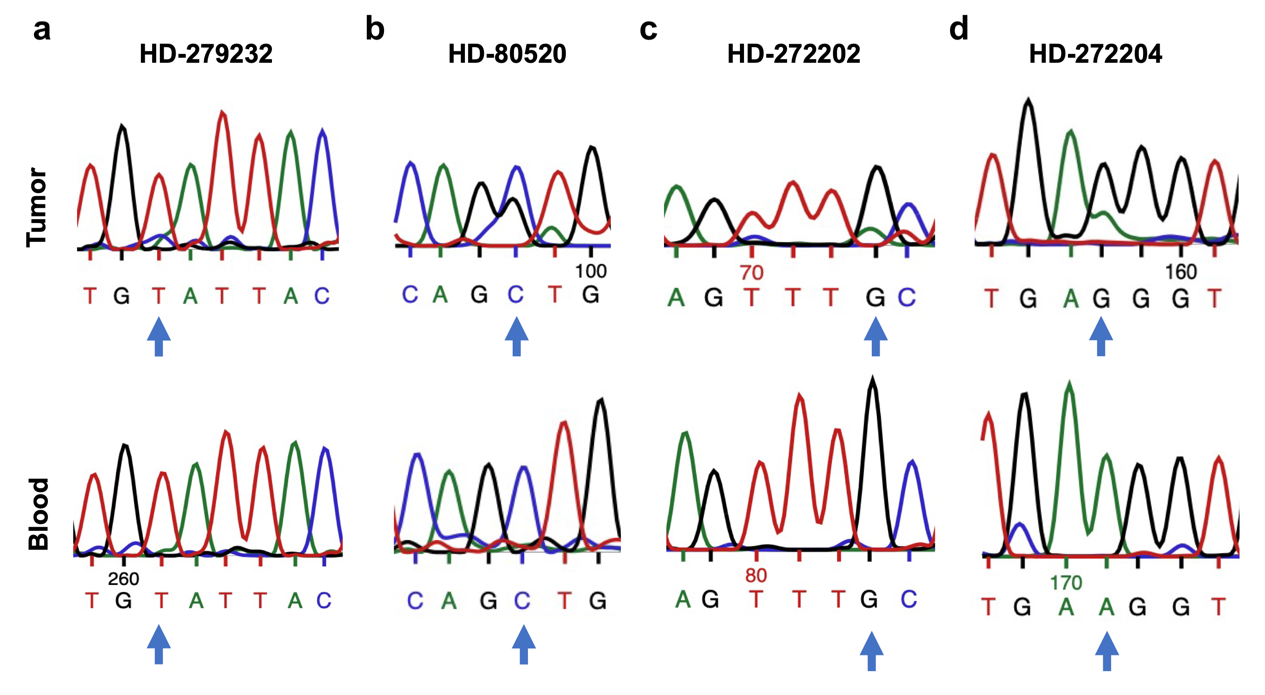
**

**Legend:** Sanger sequencing results from tumor and blood (germline) specimen are shown. Samples without a corresponding germline control were matched to artificial blood controls from our study that were predicted to be wildtype based on WES. Sample identifiers and variant positions (arrows) are indicated. **a** IGHV1-69:c.T337C:p.Y113H; **b** IGHV1-69:c.C162G:p.S54R; **c** IGHV1-69:c.G308A:p.S103N; **d** IGHV1-69:cT142C:p.F48L.

**Supplementary Fig. 8.** NOTCH1 variants – Sanger sequencing

**
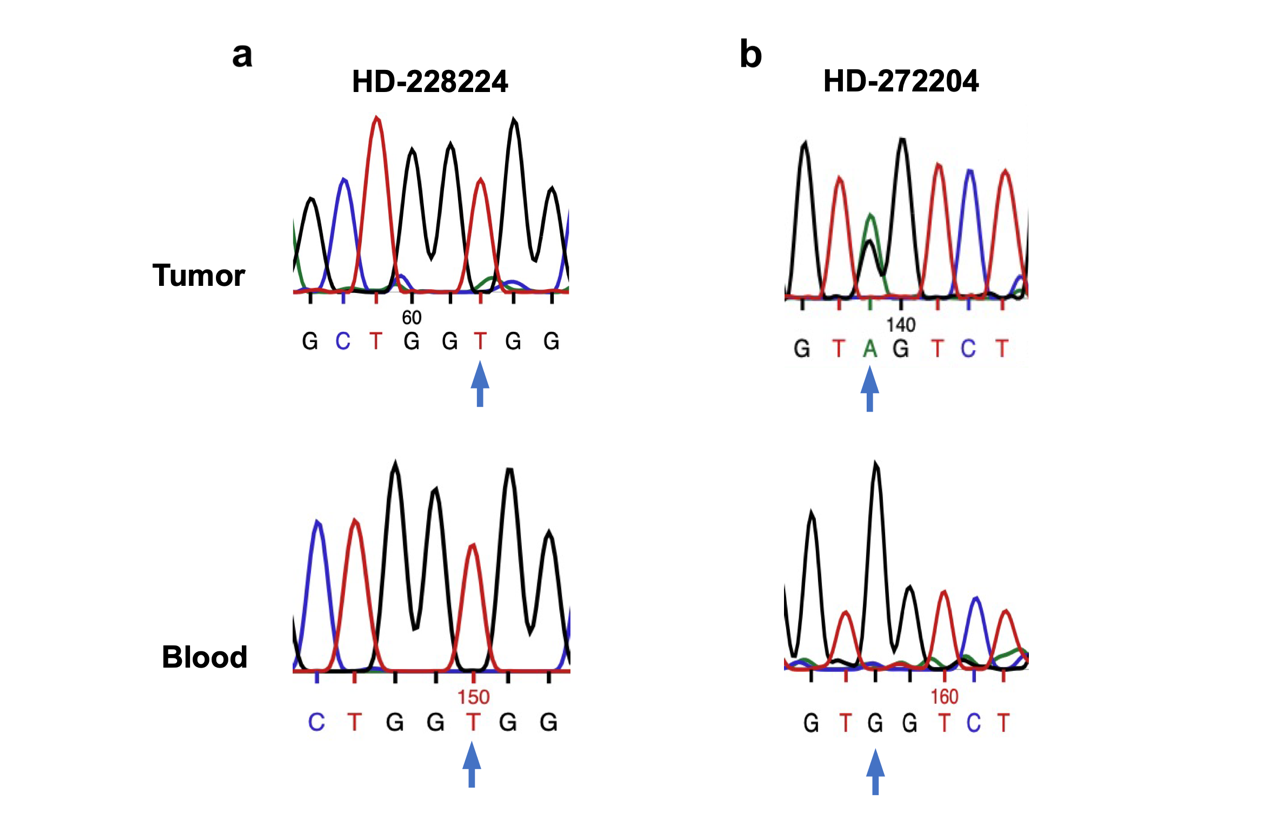
**

**Legend:** Sanger sequencing results from tumor and blood (germline) specimen are shown. Samples without a corresponding germline control were matched to artificial blood controls from our study that were predicted to be wildtype based on WES. Sample identifiers and variant positions (arrows) are indicated. **a** NOTCH1:c.A7499T:p.H2500L; **b** NOTCH1:c.C5518T:p.H1840Y.

**Supplementary Fig. 9.** KMT2D variants – Sanger sequencing

**
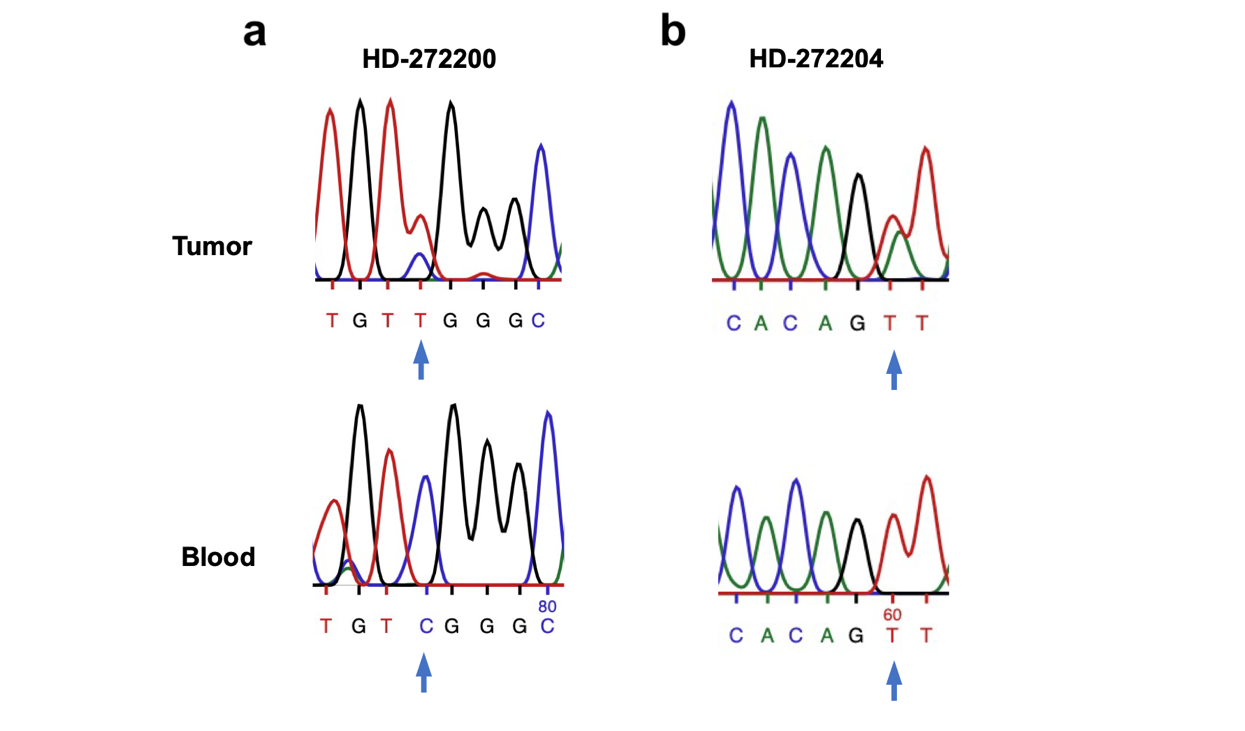
**

**Legend:** Sanger sequencing results from tumor and blood (germline) specimen are shown. Samples without a corresponding germline control were matched to artificial blood controls from our study that were predicted to be wildtype based on WES. Sample identifiers and variant positions (arrows) are indicated. **a** KMT2D:c.G9962A:p.R3321Q; **b** KMT2D:c.A16439T:p.N5480I.

**Supplementary Fig. 10.** Validation of NOTCH1 copy number gains.

**Legend:** Quantitative genomic PCR (qPCR) determined NOTCH1 copy numbers, which were normalized with GAPDH copies. Significant CNV gains were confirmed for samples, where exome sequencing revealed NOTCH1 gains (red). Two lymphoma samples without NOTCH1 gains are shown for comparison alongside matched (YNHH-6) and artificial blood controls (HD-279232). Significantly more copies (p < 0.001, Kruskal-Wallis test) were detected in tumors with NOTCH1 copy number gains based on exome sequencing data compared to blood and other unaltered tumor controls.

**Supplementary Fig. 11:** qPCR validation of selected genes from the RNASeq dataset

**Legend:** Relative gene expression normalized to GAPDH. qPCR confirmed expression of genes detected using RNASeq. In agreement with the RNASeq dataset CD163 expression, a marker for M2-macrophages, was highly expressed.

**Supplementary Methods**

**Antibodies for immunohistochemistry**

Immunohistochemistry protocols and dilutions provided by the manufacturers were followed. Antibodies are summarized in **Supplementary Table 2.**

**Supplementary Table 2**: Antibodies for immunohistochemistry

| Target gene | Antibody Clone | Species/clonality | Manufacturer | Catalogue Number |
| --- | --- | --- | --- | --- |
| CD20 | L26 | Mouse monoclonal | Dako Agilent, Santa Clara, USA | M075501-2 |
| CD10 | SP67 | Rabbit monoclonal | Ventana-Roche, Basel, Switzerland | 05857856001 |
| BCL2 | SP66 | Mouse monoclonal | Ventana-Roche, Basel, Switzerland | 06446329001 |
| BCL6 | GI191E/A8 | Mouse monoclonal | Ventana-Roche, Basel, Switzerland | 05269008001 |
| MUM1/IRF4 | MUM1p | Mouse monoclonal | Dako Agilent, Santa Clara, USA | M725901-2 |
| EBV-LMP1 | CS1-4 | Mouse monoclonal | Dako Agilent, Santa Clara, USA | M089701-2 |
| PD-L1 | SP263 | Rabbit monoclonal | Ventana-Roche, Basel, Switzerland | 790-4905 |
| Ki67 | MIB1 | Mouse monoclonal | Dako Agilent, Santa Clara, USA | GA62661-2 |

**Pan-cancer Tumor Mutational Burden (TMB)**

Exonic, non-synonymous mutational counts were determined in EBV^+^-PCNSL with germline controls included in this study (n=8), and in previously reported EBV^-^-PCNSL from the ICGC-MML-Seq Consortium [4]. Data were supplemented with whole exome sequencing (WES) data from 32 TCGA entities, which were acquired by using the R package curatedTCGAData (version 2.01) [<https://doi.org/10.1200/CCI.19.00119>]. The TMB of each sample was estimated as previously described [1, 3, 5]. Briefly, the numbers of exonic, non-synonymous mutations were divided by the exome size of 30 MB. In the following step, the median TMB was estimated for each entity and used for ranking and ordering of different tumor entities.

**Sanger Sequencing**

To confirm selected variants detected with WES we designed two PCR primers bracketing the respective variant sites with amplicons ranging between 100-250 nucleotides. Additionally, an internal primer (either forward or reverse) was designed and synthesized and used for a nested PCR (**Supplementary Table 3**). PCR products were submitted to Sanger sequencing (Eurofins Genomics, Ebersberg, Germany) with primers previously used for PCR amplification. Recommended protocols provided by Eurofins Genomics were followed. Samples without a corresponding germline control were matched to artificial blood controls from our study that were predicted to be wildtype based on exome sequencing.

PCR reactions were performed using Q5 High-Fidelity 2x Master Mix (New England Biolabs (NEB), Ipswich, USA). Per reaction 20ng of genomic DNA were used in 25 μl reaction volumes following protocols provided by the manufacturer. Briefly, cycling conditions were: initial denaturation 30 s / 98 °C; 35 cycles, denaturing 10 s / 98 °C, annealing 15 s / calculated annealing temperature, extension 20 s / 72 °C; final extension 5 min / 72 °C. The annealing temperature was determined using the NEB Tm Calculator (<https://tmcalculator.neb.com/>).

**Supplementary Table 3**: Sanger Sequencing Primers

| Gene Name | Primer Name | Forward Primer (_F) | Reverse Primer (_R) | Nested Primer (_N) |
| --- | --- | --- | --- | --- |
| SOCS1 | SOCS_1 | AATCTGGAAGGGGAAGGAGC | CACGCACTTCCGCACATT | GAAGAGGCAGTCGAAGCTCT |
| SOCS1 | SOCS_2 | AGCTCGAAGAGGCAGTCGAA | GCTGGCCCCTTCTGTAGGAT | SOCS1_R |
| IGHV1-69 | IGH69_1 | TCACACTGTGTCTCTCGCAC | CTCTCCACAGGTGTCCAGTC | CCGTGTCCTCAGATCTCAGG |
| NOTCH1 | NOTCH_1 | CGTTTACTTGAAGGCCTCCG | CAGCTACTCCTCGCCTGTG | GAGACGTTGGAATGCGGG |
| NOTCH1 | NOTCH_2 | GCTTAGGGGAGAGAGGCAG | GGTTCTGCCTGACCTGGAC | GACATTGACGTCCATGCAGT |
| KMT2D | KMT2D_1 | ATGCCCTTGATTGGACACCA | GCATTCCCTACTGTCTGCAC | CCTCCATGCTGCCCACTTA |
| KMT2D | KMT2D_2 | CCCAGCCTAGGAATCCACAT | CATCCCTGACTCTGTCCCAC | CTCACCTCCTCTCCTTTGGG |

**qPCR – Validation of CNVs**

To confirm selected copy number variants detected with exome sequencing, previously published primers and protocols were used [2]. If primers were unavailable, they were designed with Primer BLAST (National Center for Biotechnology Information https://www.ncbi.nlm.nih.gov/tools/primer-blast/. Primer sequences are summarized in **supplementary table 4**. Briefly, to amplify only genomic DNA primers covered intron-exon junctions. Reactions were prepared with power SYBR Green PCR Master Mix (Thermo Fisher, Waltham, USA). 20ng of genomic DNA and 300nM of forward and reverse primer were used per reaction. Each tumor and corresponding blood sample was subjected to qPCR for target and reference gene (GAPDH). If matching germline controls were unavailable, tumor samples were matched to artificial same gender blood controls. Cycling conditions followed manufacturer’s protocols. The delta threshold cycle (CT) between GAPDH and target gene amplification was calculated and compared with data from blood controls.

**Supplementary Table 4**: qPCR Primers

| **Gene** | **Primer Name** | **Forward (_F)** | **Reverse (_R)** | **Source** | **Link** |
| --- | --- | --- | --- | --- | --- |
| NOTCH1 | NOTCH1 | AGCCTGCACAACCAGACAGA | GCCACTGCCTACCTGGAAGA | Katarkar et al. Nat. Comm 2020 [2] | https://www.nature.com/articles/s41467-020-18919-2#MOESM5 |
|  | NOTCH2 | CAGGGTAACCAGGGCCTTC | CCCTCGACAAAGCAACAGGT | Katarkar et al. Nat. Comm 2020 [2] | <https://www.nature.com/articles/s41467-020-18919-2#MOESM5> |
| GAPDH | GAPDH1 | CTGCCACCCAGAAGACTGTG | GGTCAGGTCCACCACTGACA | Katarkar et al. Nat. Comm 2020 [2] | <https://www.nature.com/articles/s41467-020-18919-2#MOESM5> |
|  | GAPDH2 | TGGTCTCCTCTGACTTCAACAG | AGAAGATGAAAAGAGTTGTCAGGGC | Primer BLAST | <https://www.ncbi.nlm.nih.gov/tools/primer-blast/index.cgi> |

**qPCR – Validation of RNASeq data**

Reverse transcription (RT) of extracted RNA (10ng / qPCR reaction planned) was performed with the MultiScribe Reverse Transcriptase 50U/μLkit (Applied Biosciences, Waltham, USA). Samples with sufficient leftover RNA quantities from expression clusters two and three were selected. RT was performed according to the manufacturer’s recommendations. Briefly cycler conditions were: Hold 10 mins, 25 °C; Hold 120 mins, 37 °C; Hold 5 mins, 85 °C; Infinite hold, 4 °C. For subsequent qPCR the following Taqman probes were obtained from Thermo Fisher (Waltham, USA): GAPDH, Hs99999905_m1; ACTB, Hs99999903_m1; CD70, Hs00174297_m1; IL1R2, Hs00174759_m; PD-L1/CD274, Hs00204257_m1; FOXP3, Hs01085834_m1; CD163, Hs00174705_m1. Assuming complete RT, 10ng of cDNA/reaction were subjected to qPCR, which was performed with the 2x TaqMan Gene Expression Fast Advanced Master Mix (Applied Biosystems, Waltham, USA). Cycler settings for qPCR were: Hold 2 mins, 50 °C; Hold 20 s, 95 °C; 40 Cycles: 1s / 95 °C; 20 s / 60 °C. Threshold cycles (CT) for target genes were determined and normalized with housekeeping gene expression.

**Supplementary Table 5.** Healthy brain controls from the TCGA-GTEx dataset

| **ID from this study** | **GTEx ID** | **Release** | **Localization** | **Gender** |
| --- | --- | --- | --- | --- |
| Brain control 1 | GTEX-ZVZQ-0011-R5b-SM-57WDC | GTEx dbGaP release v8 | Caudate | Female |
| Brain control 2 | GTEX-11H98-0011-R5b-SM-57WCF | GTEx dbGaP release v8 | Caudate | Male |
| Brain control 3 | GTEX-ZVZQ-0011-R7b-SM-57WBB | GTEx dbGaP release v8 | Putamen/basal ganglia | Female |
| Brain control 4 | GTEX-11NV4-0011-R7a-SM-57WDH | GTEx dbGaP release v8 | Putamen/basal ganglia | Male |
| Brain control 5 | GTEX-11OF3-3126-SM-5GU5E | GTEx dbGaP release v8 | Cerebellum | Male |
| Brain control 6 | GTEX-111FC-3126-SM-5GZZ2 | GTEx dbGaP release v8 | Cortex | Male |
| Brain control 7 | GTEX-ZYY3-3126-SM-5SI9L | GTEx dbGaP release v8 | Cortex | Female |
| Abbreviations: ID, identifier; GTEx, the Genotype Tissue Expression Portal; v, version | | | | |

**Supplementary References**

1. Chang H, Sasson A, Srinivasan S, Golhar R, Greenawalt DM, Geese WJ, Green G, Zerba K, Kirov S, Szustakowski J (2019) Bioinformatic Methods and Bridging of Assay Results for Reliable Tumor Mutational Burden Assessment in Non-Small-Cell Lung Cancer. Mol Diagn Ther 23: 507-520. https://doi.org/10.1007/s40291-019-00408-y

2. Katarkar A, Bottoni G, Clocchiatti A, Goruppi S, Bordignon P, Lazzaroni F, Gregnanin I, Ostano P, Neel V, Dotto GP (2020) NOTCH1 gene amplification promotes expansion of Cancer Associated Fibroblast populations in human skin. Nat Commun 11: 5126. <https://doi.org/10.1038/s41467-020-18919-2>

3. Mankor JM, Paats MS, Groenendijk FH, Roepman P, Dinjens WNM, Dubbink HJ, Sleijfer S, Cuppen E, Lolkema MPJK, Consortium C (2020) Impact of panel design and cut-off on tumour mutational burden assessment in metastatic solid tumour samples. <https://doi.org/10.1038/s41416-020-0762-5>

4. Radke J, Ishaque N, Koll R, Gu Z, Schumann E, Sieverling L, Uhrig S, Hübschmann D, Toprak UH, López C et al (2022) The genomic and transcriptional landscape of primary central nervous system lymphoma. Nat Commun 13: 2558. <https://doi.org/10.1038/s41467-022-30050-y>

5. Sha D, Jin Z, Budczies J, Kluck K, Stenzinger A, Sinicrope FA (2020) Tumor Mutational Burden as a Predictive Biomarker in Solid Tumors. Cancer Discov 10: 1808-1825. https://doi.org/10.1158/2159-8290.Cd-20-0522
